# Dramatic transcriptomic differences in *Macaca mulatta* and *Macaca fascicularis* with *Plasmodium knowlesi* infections

**DOI:** 10.1038/s41598-021-98024-6

**Published:** 2021-09-30

**Authors:** Anuj Gupta, Mark P. Styczynski, Mary R. Galinski, Eberhard O. Voit, Luis L. Fonseca

**Affiliations:** 1 The Wallace H. Coulter Department of Biomedical Engineering, Georgia Institute of Technology and Emory University, Atlanta, GA USA; 2 School of Chemical & Biomolecular Engineering, Georgia Institute of Technology, Atlanta, GA USA; 3 Emory Vaccine Center, Yerkes National Primate Research Center, Emory University, Atlanta, GA USA; 4 Division of Infectious Diseases, Department of Medicine, Emory University, Atlanta, GA USA; 5 Laboratory for Systems Medicine, Department of Medicine, University of Florida, Gainesville, FL USA

**Keywords:** Computational biology and bioinformatics, Diseases, Medical research, Pathogenesis

## Abstract

*Plasmodium knowlesi,* a model malaria parasite, is responsible for a significant portion of zoonotic malaria cases in Southeast Asia and must be controlled to avoid disease severity and fatalities. However, little is known about the host-parasite interactions and molecular mechanisms in play during the course of *P. knowlesi* malaria infections, which also may be relevant across *Plasmodium* species. Here we contrast *P. knowlesi* sporozoite-initiated infections in *Macaca mulatta* and *Macaca fascicularis* using whole blood RNA-sequencing and transcriptomic analysis. These macaque hosts are evolutionarily close, yet malaria-naïve *M. mulatta* will succumb to blood-stage infection without treatment, whereas malaria-naïve *M. fascicularis* controls parasitemia without treatment. This comparative analysis reveals transcriptomic differences as early as the liver phase of infection, in the form of signaling pathways that are activated in *M. fascicularis*, but not *M. mulatta*. Additionally, while most immune responses are initially similar during the acute stage of the blood infection, significant differences arise subsequently. The observed differences point to prolonged inflammation and anti-inflammatory effects of IL10 in *M. mulatta*, while *M. fascicularis* undergoes a transcriptional makeover towards cell proliferation, consistent with its recovery. Together, these findings suggest that timely detection of *P. knowlesi* in *M. fascicularis*, coupled with control of inflammation while initiating the replenishment of key cell populations, helps contain the infection. Overall, this study points to specific genes and pathways that could be investigated as a basis for new drug targets that support recovery from acute malaria.

## Introduction

Malaria has plagued humanity since the dawn of civilization and is one of the world’s deadliest infectious diseases, with an estimated 229 million cases and 409,000 deaths reported in 2019^1,2^. Malaria has been studied scientifically since the late 1800’s, when the disease was blamed on “bad air” (Italian: *mal’ aria*), and great progress has been made toward understanding and treating the disease since the identification of the infecting agent as mosquito-borne *Plasmodium* parasites^3–5^. Yet, malaria still persists throughout the world, including with zoonotic pathogens and increased resistance to anti-malarial drugs^6,7^. Fundamental studies of the basic mechanisms of *Plasmodium* infections in the liver, and subsequently in the blood, and their effects on the host continue to be direly needed.

Numerous cross-sectional blood sample examinations from individuals with varying levels of malarial disease have been carried out around the world for decades to diagnose individuals and conduct research^8^, however, the current investigative repertoire for studying the temporal changes associated with disease progression in humans is relatively scarce^9–11^. The main reason is of an ethical nature, typically mandating the treatment of patients as soon as they are diagnosed. Consequently, the main source of blood samples for research on natural infections has been from active case detection and symptomatic individuals seeking treatment. It is evident that knowledge of malaria and its progression concurrently with immune responses developing within a host is a prerequisite for rationally developing new measures for preventing the disease, treating patients, and improving patient outcomes. While immunity and vaccine research is advancing in the context of controlled human malaria infection studies initiated with sporozoites^12–20^, performing these longitudinal molecular studies with human volunteers has its logistical and ethical challenges^21^, and immediate treatment of blood-stage infections has been a standard requirement.

Rodent malaria models have been widely used to expand our understanding of *Plasmodium* infections^22–24^. While they offer a spectrum of advantages, the differences in human and mouse or rat physiology present some drawbacks. Nonhuman primate (NHP) macaque models are much closer to humans, and the clinical presentation of malaria and immune responses are more similar between humans and macaques^25–29^. As a consequence, macaques have become important alternatives to rodent models for explaining different host–pathogen interactions, not only for malaria, but also for various diseases including those caused by HIV/SIV and COVID^30,31^. Indeed, infection of macaques are widely accepted as robust in vivo models for human malaria, with comparable liver- and blood-stage cycles^32,33^.

Between 2012 and 2019, under the auspices of research contracts from the U.S. National Institute of Allergy and Infectious Diseases (NIAID) and the U.S. Defense Advanced Research Projects Agency (DARPA), we collected and deposited in NIAID-supported Bioinformatics Resource Centers (BRCs) large clinical, parasitological, immunological and multi-omic datasets from longitudinal infections of macaque species infected with *Plasmodium coatneyi*, *Plasmodium cynomolgi* or *Plasmodium knowlesi*^34,35^*.* Our investigations yielded a large collection of datasets and analyses from over 30 macaque infections and controls, including frequent samplings of blood and bone marrow^34,36–43^. The first two simian malaria parasite species are excellent models of corresponding human infections by the parasites *P. falciparum* and *P. vivax*, respectively^28,44^, while *P. knowlesi* reflects aspects of both, depending on the question^29,45^. *Plasmodium cynomolgi* and *P. knowlesi* infect both humans and NHPs and constitute zoonotic public health concerns in Southeast Asia, where humans and macaque species coexist^7,46–49^. Comparative longitudinal macaque infection studies of the type performed by our consortium can help focus analyses on significant molecular features that play crucial roles in determining the course of the disease. Furthermore, because host–pathogen interactions in macaques can closely mimic those in humans, findings from macaques have great potential for establishing a rational basis for new therapeutic targets and interventions, including host-directed therapies.

For the analyses described here, we used peripheral blood transcriptomics data from cohorts of *Macaca mulatta* (Mm) and *Macaca fascicularis* (Mf), which were infected with sporozoites of the Malayan strain of *P. knowlesi*^50^. Here we study host responses prior to and from the time of parasite inoculation through the development of liver-stage schizonts containing invasive merozoites followed by the release of these merozoites from infected hepatocytes into the bloodstream. Cyclical merozoite invasion and multiplication within red blood cells (RBCs) caused the progression of malarial illness and disease manifestations, as presented elsewhere^51^.

While Mm and Mf are closely related NHP species (Figure S1) with reported interbreeding and shared geographical locations^52^, an important difference must be noted. Namely, Mf co-evolved with *P. knowlesi* within a large geographical area of Southeast Asia, whereas the distribution of Mm overlaps with *P. knowlesi* only slightly (Figure S2). Arguably as a consequence, Mf shows signs of the disease but survives with a low-level infection that can become chronic, whereas Mm becomes severely ill with escalating life-threatening parasitemia and succumbs unless treated^32,33,53,54^. The molecular and physiological basis of this stark difference is unknown. We found that the peripheral blood transcriptional responses have many similarities between the two species, but subtle differences are significant with regard to the observed outcomes. In particular, our analysis suggests that Mf initiates its transcriptomic response earlier than Mm and with some favorable adjustments around the time of the acute blood-stage infection that could contribute to the control of parasitemia and help enable the recovery of Mf.

## Results

**Figure 1:**
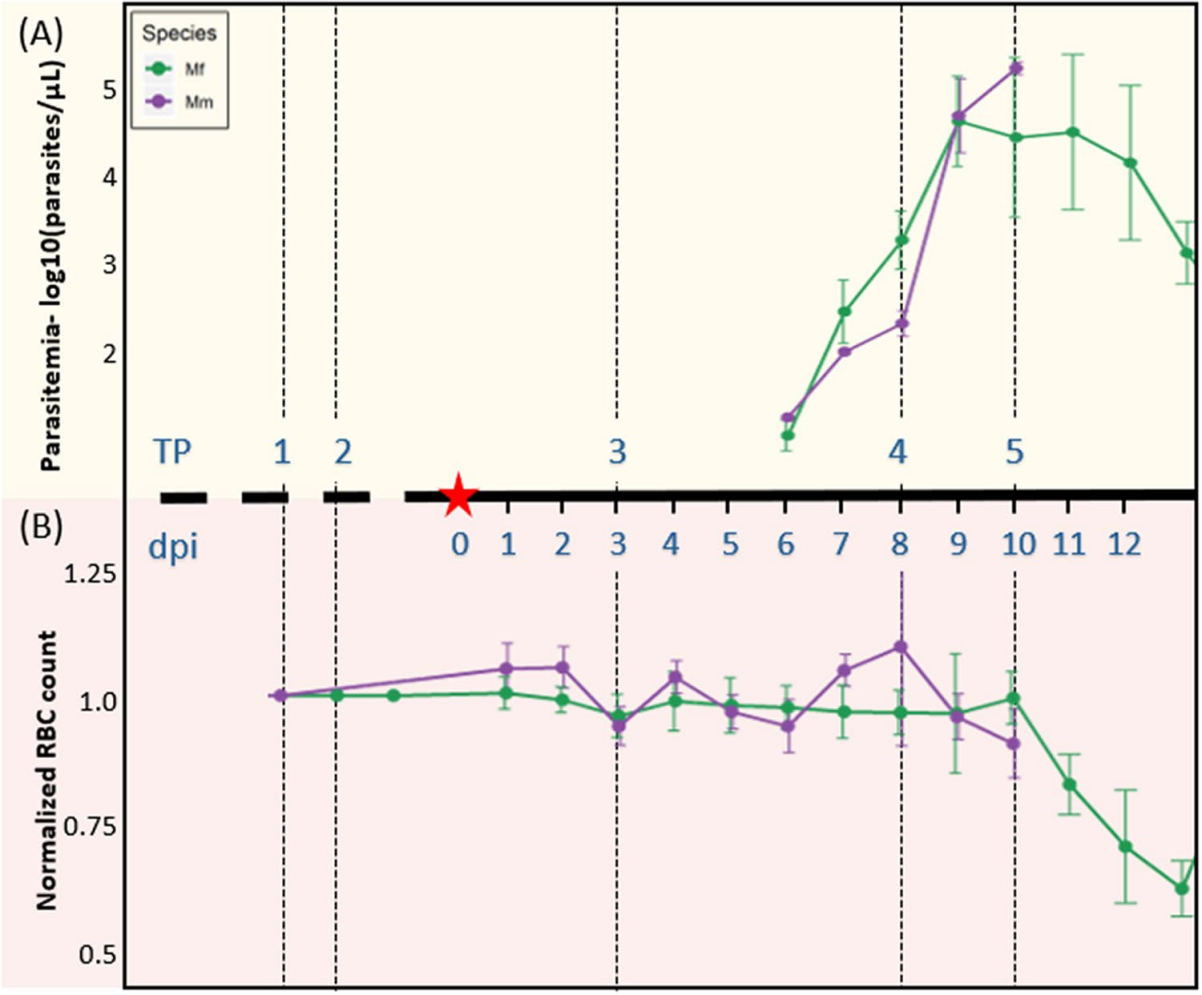
Timeline of Mm and Mf infection with *P. knowlesi*, along with parasitemia levels and RBC counts. The x-axis depicts the time points (TPs) and days post inoculation (dpi). The red star represents the day of sporozoite inoculation (i.e*.*, dpi 0). Data at TP1 were collected more than a month before TP2. (**A**) Parasitemia: The y-axis shows average parasitemia levels throughout the *P. knowlesi* infection on a log10 scale. Parasitemia levels were measured as parasites/μL. (**B**) Normalized RBC counts: The y-axis shows the ratio of mean RBC counts with respect to pre-infection levels**.**

The results of our analysis are based on longitudinal transcriptomics data from whole blood samples taken at five time points (TPs) from cohorts of four Mm and seven Mf. The subjects were infected sequentially at different times by inoculation with the same batch of cryopreserved *P. knowlesi* sporozoites (Fig. 1). All data generated from these infections are available in public databases and details of the experimental design and data deposition are discussed in the Methods section. The overall goal here is to compare and characterize the temporal whole blood transcriptional programs launched by the two macaque species in response to the infection. Analyses of other datasets and their integration in the context of this experiment will be discussed elsewhere (manuscripts in preparation).

### *Plasmodium knowlesi* infection causes different gene expression patterns in *Macaca mulatta* (Mm) and *Macaca fascicularis* (Mf)

**Figure 2:**
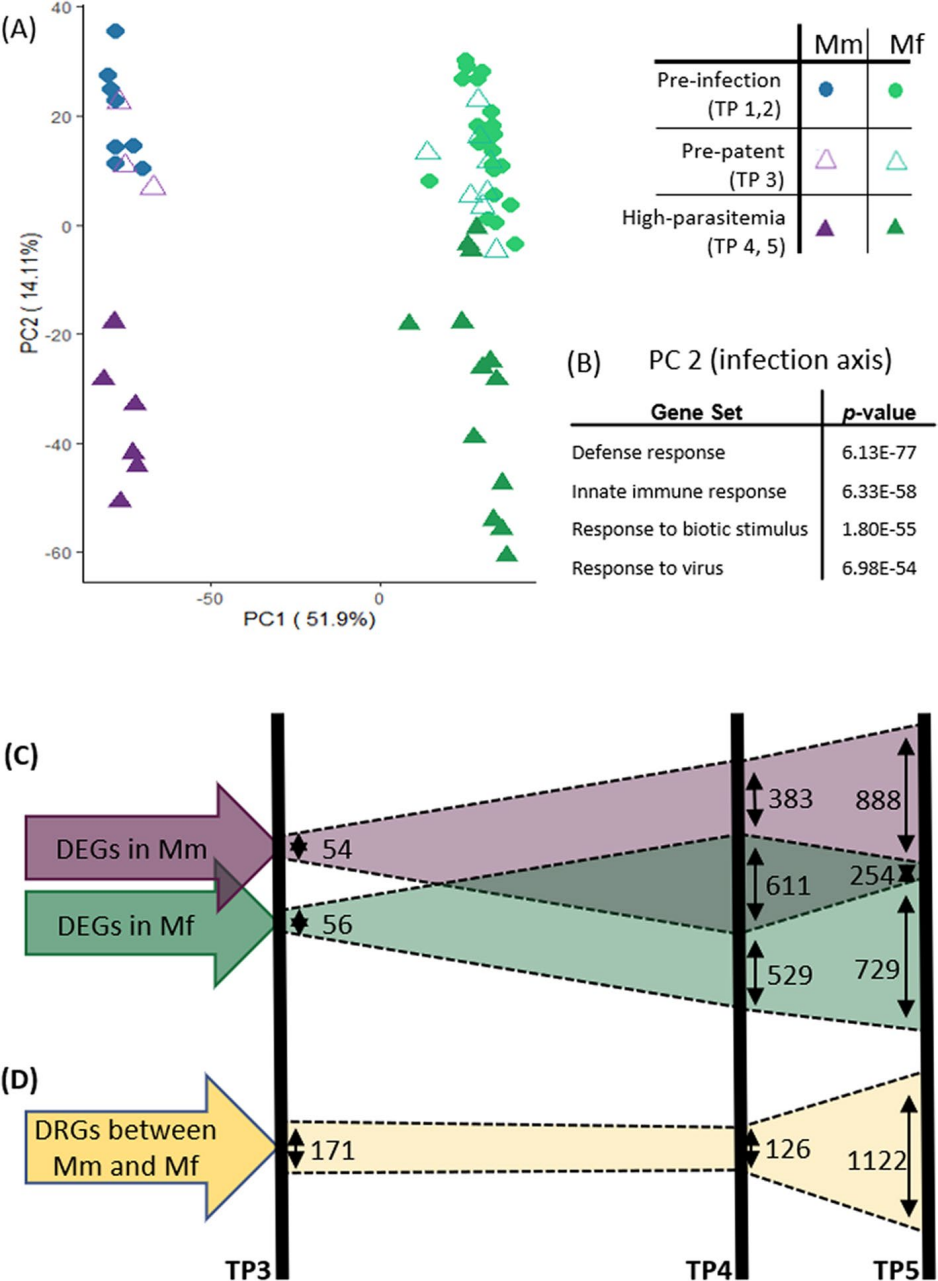
Transcriptomics patterns of whole-blood gene expression of all samples at time points TP1–TP5 from Mm and Mf. (**A**) Principal component analysis (PCA) of all samples from Mm and Mf. PC1 captures inter-species variance in expression profiles, while PC2 captures temporal variance in expression profiles. Pre-infection samples TP1 and TP2 (before infection) form species-specific clusters that are separate from the samples at TP4 and TP5, reflecting peaking parasitemia. TP3 (3 days after inoculation of sporozoites, and prior to blood-stage parasitemia, *i.e*., the pre-patent period) samples cluster with the pre-infection samples TP1 and TP2. (**B**) Top GO gene sets over-represented in PC2. (**C**) Differentially expressed genes (DEGs) and (**D**) Differentially responding genes (DRGs) in TP3-TP5. The numbers of DEGs change significantly between TP3 (~ 50) and TP4 (~ 1000–1140) for both Mm (purple) and Mf (green), and then remain similar between TP4 and TP5 (~ 1000–1100) for each species. However, the number of common DEGs between Mm and Mf decreases substantially from TP4 (611) to TP5 (254). Concomitantly, the DRGs between TP4 and TP5 (gold) increase substantially.

As expected, gene expression repertoires and levels changed in both monkey species during the course of experimentally introduced *P. knowlesi* sporozoite infections, both as the *P. knowlesi* parasites multiplied within hepatocytes and following their cyclical replication in host RBCs and parasitemia patency (Fig. 1A, 6 days post inoculation “dpi” onwards). To characterize these changes, we compared RNA-seq data generated from samples acquired from both macaque species at baseline and at specific infection time points (TPs). Principal component analysis (PCA) of the whole-blood gene expression patterns (Fig. 2A) shows a clear separation of the two species, as well as between samples taken before infection (TP1 and TP2) and during the fast rise and approach of peaking parasitemia (TP4 and TP5, respectively 8 and 10 dpi). Samples taken shortly after the inoculation of sporozoites, during the pre-patent period (TP3, 3 dpi), cluster mostly with pre-infection samples. Most of the variance (PC1 = 51.9%) shown is due to the host species, while the second major axis of variance (PC2 = 14.11%) separates pre-infection (TP1, TP2) from acute-parasitemia samples (TP4, TP5). Analysis of pre-infection TP1 and TP2 samples with pre-patent TP3 samples (Figure S3) demonstrates dominance of inter-individual variability over short-term transcriptomic changes during the initial phase of the infection.

It is worth noting that the same PC2 axis crisply separates pre-infection (TP1, TP2) and acute infection (TP4, TP5) samples for both host species even though the disease progression is different, suggesting fundamentally similar transcriptional responses. To gain deeper insights into the details of PC2, we identified enriched Gene Ontology (GO) gene sets along this axis (Fig. 2B). The most enriched gene sets are associated with defense and innate immune responses (*p* ≈ 10^−70^) and with a response to cytokine and biotic stimuli (Figure S4).

To characterize key features in response to the *P. knowlesi* infection in each host species, we identified significant intra-species and inter-species transcriptional changes. For intra-species analyses, we identified differentially expressed genes (DEGs) for each infection time point (TP3, TP4 and TP5) compared to the pre-infection baseline samples (TP1 and TP2). For inter-species analyses, we identified genes that responded differentially to infection, as explained further in our supplementary data (Figure S5). These differentially responding genes (DRGs) provided contrasting differences in infection responses between the species at each time point.

As early as TP3, when the infection was still confined to the liver, some changes in blood transcriptome were identified in each host species. These are statistically significant, although they are quantitatively much smaller than the changes that occur when parasitemia is rising and peaking (TP4 and TP5, respectively). While many DEGs are shared between the two species at TP4, there is greater divergence in the transcriptional profiles at TP5, as indicated by fewer shared DEGs (Fig. 2C). This divergence is supported by the results of our inter-species analyses, where we observe only a few DRGs up to TP4, but a substantial increase in DRGs at TP5 (Fig. 2D).

### Evolutionary distance of homologous genes does not account for the differential responses observed in the *Macaca mulatta* (Mm) and *Macaca fascicularis* (Mf) host species

We hypothesized that there might be a relationship between the evolutionary divergence of homologous Mm and Mf genes and their expression profiles in the two hosts, since such divergence could be explained by evolutionary pressure and possibly underpin the differences in the control of parasitemia and in the different outcomes observed between the species. To test this hypothesis, we estimated the evolutionary distance between homologous genes in the two species (Table S9) and compared the similarity scores of DRGs at each time point.

**Figure 3:**
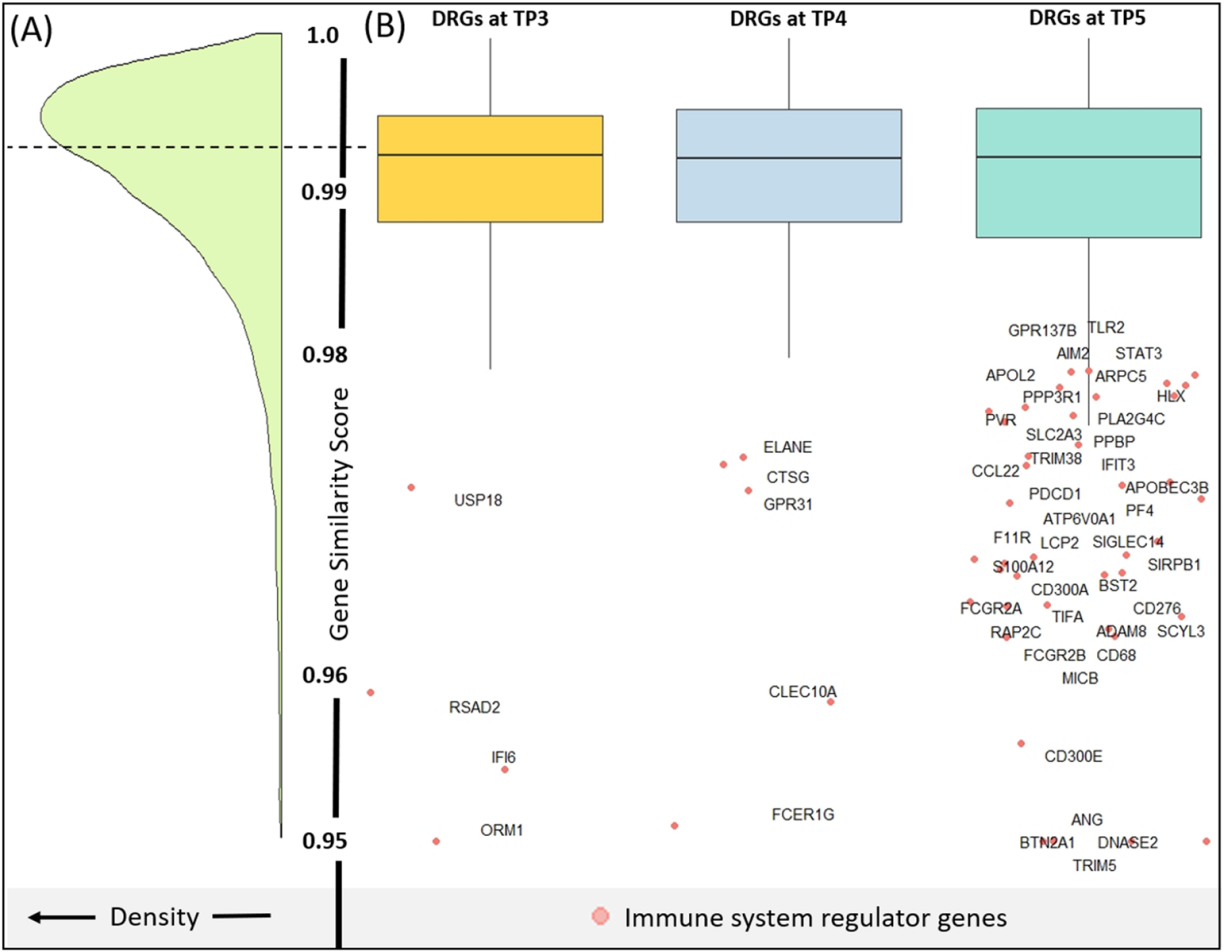
(**A**) Density plot showing the distribution of genes across similarity score of ~ 15,000 homologous genes. (**B**) Box plot comparing the gene similarity score of DRGs at TP3, TP4 and TP5. Although the mean similarity score across DRGs at TP4 and TP5 is not different as compared to all genes, GO annotation of the outlier genes (Red points) suggests numerous genes being involved with immune regulation.

The evolutionary hypothesis was ultimately rejected given a gene similarity score of all genes that was not significantly different (Kolmogorov–Smirnov *p-*value > 0.1) from that of the three sets of DRGs (Fig. 3). However, it is interesting to note that genes involved in regulation of immune system processes were overrepresented in the outliers at each time point, with a hypergeometric *p*-value of 3.9 × 10^−18^ and 3.29-fold over-enrichment in the outliers compared to expectation in all immune system genes. Of particular interest are immune system related genes (FCER1G and ELANE), cytokines and growth factors (CCL22, CTSG, PF4 and PPBP), transcription factors (STAT3, TRIM38 and HLX) and various cell differentiation markers (BST2, CD276, CD300A, CD68, CLEC10A, F11R, FCGR2A, FCGR2B, PDCD1, PVR, SIRPB1 and TLR2).

### Gene set enrichment analysis indicates distinctive gene expression profiles between the species by TP5

To identify well-defined, significantly enriched gene sets with respect to *P. knowlesi* infection, we performed gene set enrichment analysis (GSEA) using significant DEGs (adjusted *p* < 0.01, log2 (absolute fold change) > 1) at each time point. We used Hallmark gene sets from the Molecular Signature database^55^ for an overview analysis (Table S1) followed by a detailed analysis using GO annotated biological pathways. Intriguingly, by TP3 a few DEGs were identified that only showed significant enrichment in the Mf. Although the number of DEGs is similar in both species, Mf has several Hallmark gene sets significantly enriched (Table S1). Importantly, these results suggest that by 3 dpi, when parasites solely reside in the liver, Mf is already mounting interferon (IFN)-mediated immune responses (IFNα and IFNγ) against the parasite. Relevant genes involved in the response include IRF7, CCL22, CXCL12 and PML. In-depth GO analysis (Figure S6) indicates that the IFN responses are characterized by pathways known to regulate viral genome replication and the cytoplasmic pattern recognition receptor (PRR) signaling pathway, known to indicate the presence of foreign genetic material. Differences between the two species are less evident by TP4, when parasitemia is rising. Then, both species show pronounced enrichment for IFNα and IFNγ immune responses and several signaling pathways, including NFκB and IL6-JAK-STAT3 (Figure S7, Table S1).

In-depth GO analysis shows that while many aspects of the response at TP4 are similar for the two species, including the typical response to viruses, Type-1 IFN production, and urea catabolism, some notable differences exist (Figure S8). For instance, Mm exhibits prominent regulation of calcium ion transport along with changes in certain metabolic pathways including amino acid metabolism, protein catabolism and cytokine metabolism. In Mf, by contrast, cellular amide metabolic processes (urea catabolism) and additional immune response pathways triggered by foreign organisms dominate the response. Interestingly, Mf also shows enrichment of an adaptive immune pathway.

**Figure 4:**
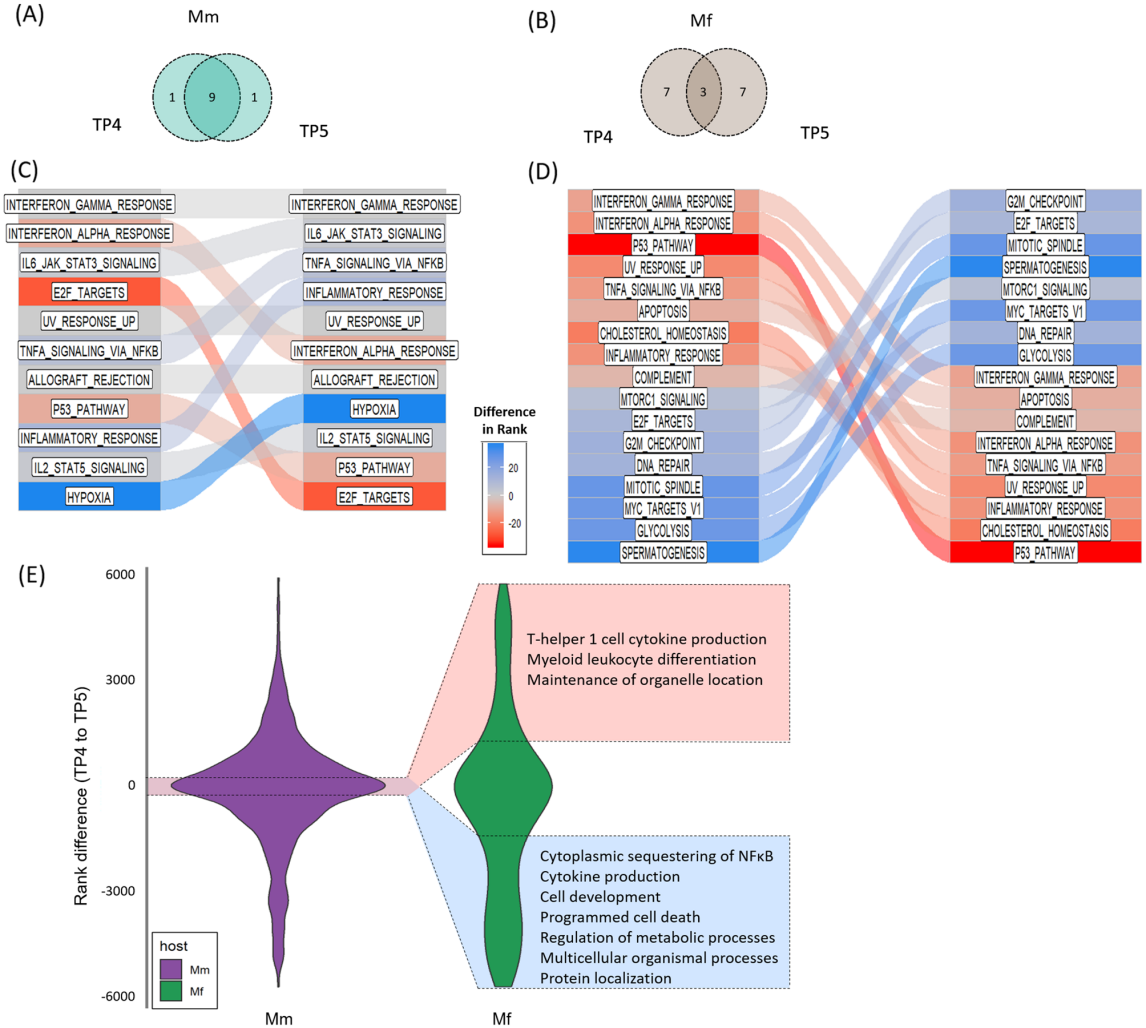
Changes in enriched pathways from TP4 to TP5 in Mm and Mf. (**A**, **B**): Venn diagrams of top 10 enriched pathways at TP 4 and TP5 for Mm and Mf, respectively. In Mm, 9 out of the top 10 are common between the two TPs. In Mf, only 3 out of the top 10 are common. (**C**, **D**): Ranking, from top to bottom, of most enriched pathways in Mm and Mf, respectively, at TP4 and TP5 (left to right). For each host, the changes in ranks of the top pathways between TP4 to TP5 are shown. Each list contains the union of the most significantly changed gene sets, ordered by their enrichment score. The top ranked gene set is the most enriched. Connectors show changes in rank for each gene set between the two TPs. Red shading indicates increases in rank and hence decreased enrichment from left (TP4) to right (TP5). Blue shading indicates decreases in rank and thus increased enrichment from TP4 to TP5. Color shades are proportional to relative changes in rank considering all gene sets (not just the ones shown). For instance, the darker shade of red for p53 pathway in panel D represents a sharp decrease in rank. (**E**): Violin plots of rank differences, representing the importance of gene sets, between TP4 and TP5 in the two host species. The vertical axis represents the distribution of rank differences between TP4 and TP5 for GO gene sets in Mm and Mf. Each data point in the two distributions represents one GO gene set (see Methods for details). The transition from TP4 to TP5 in Mm is characterized by a distribution (shown in purple) with 0 mean and a broad, rather than narrow distribution, which corresponds to relatively small changes in the importance of most gene sets. By contrast, the distribution of ranks in Mf (shown in green) has a much narrower distribution at the mean, with heavy tails, indicating many more changes in rank, overall. Both red and blue domains represent pathways that are important in Mm during both TP4 and TP5. In Mf, the red domain represents pathways that were not important at TP4 (higher ranked/lower enrichment) but become more important at TP5 (lower ranked/higher enrichment), while the blue domain represents pathways that were important at TP4 but become less important at TP5. The striking difference between these distributions demonstrates that Mf alters and refocuses its gene expression profile between TP4 and TP5 towards cell proliferation, etc. In contrast, Mm’s gene expression remains almost unchanged, still emphasizing the immune response.

At TP5, which is only 2 days later than TP4 (Fig. 1A), the differences between the species in their most significantly enriched pathways become much more profound (Figs. 4, S7, S9; Table S1). Mm continues to express mainly immune response genes related to cytokine secretion, leukocyte activation and responses to the presence of foreign organisms, while the Mf gene expression profile shifts dramatically.

Interestingly, some of the adaptive immune pathways previously only identified in Mf at TP4 (Figures S8) are evident in Mm at TP5 (Figure S9). Beyond these differences, the two hosts have some pathways in common at TP5, mostly associated with immune responses to inflammation and metabolic pathways regulating cell cycle and protein modification.

Upon further analysis of the differences between TP4 and TP5 (Fig. 4), a dramatic contrast emerges: the response of Mm at TP4 and TP5 is very similar, whereas Mf shifts into a different phase of its response after TP4, characterized by what seems like an effort towards rehabilitation and recovery. The immune responses in Mf are still evident at TP5, but gene expression in this species notably shifts toward cell proliferation and cell division functions, highlighted by DNA replication, chromosome segregation, organelle fission and localization pathways. One of the noticeable enrichment changes in Mf is in the p53 pathway (Fig. 4D), with the timely enrichment of this stress response pathway by TP4, and its ceased enrichment by TP5. This observation can be regarded as a precursor for DNA replication, cell division and cell proliferation pathways^56^. Moreover, we were able to extract more information about previously identified cell cycle related pathways (Fig. 4E). Myeloid leukocyte differentiation hints at upregulation of specific cell populations in Mf. It is worth noticing that Mf downregulates cytokine production while upregulating Th1 cell cytokine production, which is a part of the adaptive immune response. Enrichment of the JAK-STAT pathway (Figure S7) in Mm along with its cross-regulation with both IL10 and IL6 suggests duplicated efforts^57^. Differential expression of the SOCS3 gene in both Mm and Mf suggests upregulation of the JAK-STAT pathway but the two-fold regulation could mean impaired functionality in Mm. Overall, as discussed further below, the GSEA reveals a prominent difference in molecular responses that might be responsible for the diverse outcomes of the two macaque hosts.

In sum, Mf detects the pathogen sooner than Mm and is able to balance its immune response and inflammation in the face of higher parasitemia levels. This earlier response is consistent with the conclusions of Peterson et al*.*^51^ from their analysis of clinical, parasitological and immune response data for these infected animals. Interestingly, in Mm, a strong enrichment of Ca^2+^ ion transport might be playing an important role in pathogen survival as it maintains Ca^2+^ homeostasis and aids the parasite’s Ca^2+^-based signaling, which is critical for parasite growth and differentiation within infected RBCs, and their egress and invasion of new host RBCs during the blood stage of the disease^58^. The transition from TP4 to TP5 highlights key differences in immune responses between the two species. Gene expression at TP4 has significant similarities between the two species, although there are some key differences. Then the species’ responses diverge. Figures 4, S7 and S10 demonstrate that while Mm does not seem to change its response much, Mf mounts a radical makeover in expression profiles in important pathways between TP4 and TP5, and these changes appear to be among the most crucial differences observed between the two species. In contrast to Mf’s response, Mm displays fairly small changes among the most enriched gene sets during the transition from TP4 to TP5, exceptions being an increased focus on hypoxia-related genes and decreased importance of E2F target genes at TP5.

### Analysis of transcription factors reveals prominent regulators that distinguish the immune response of the two hosts

To characterize these differences further, we identified the transcription factors (TFs) and other regulatory proteins that might orchestrate changes in these coordinated gene programs. These TFs and the gene networks they control can be found by searching for upstream regulators of DEGs in the databases iRegulon^59^ and TRRUST DB^60^ (see “Methods”). The most significantly enriched TFs, according to a combination of the two databases, are shown in Fig. 4A; an exhaustive list of iRegulon TFs identified is presented in Table S3.

**Figure 5:**
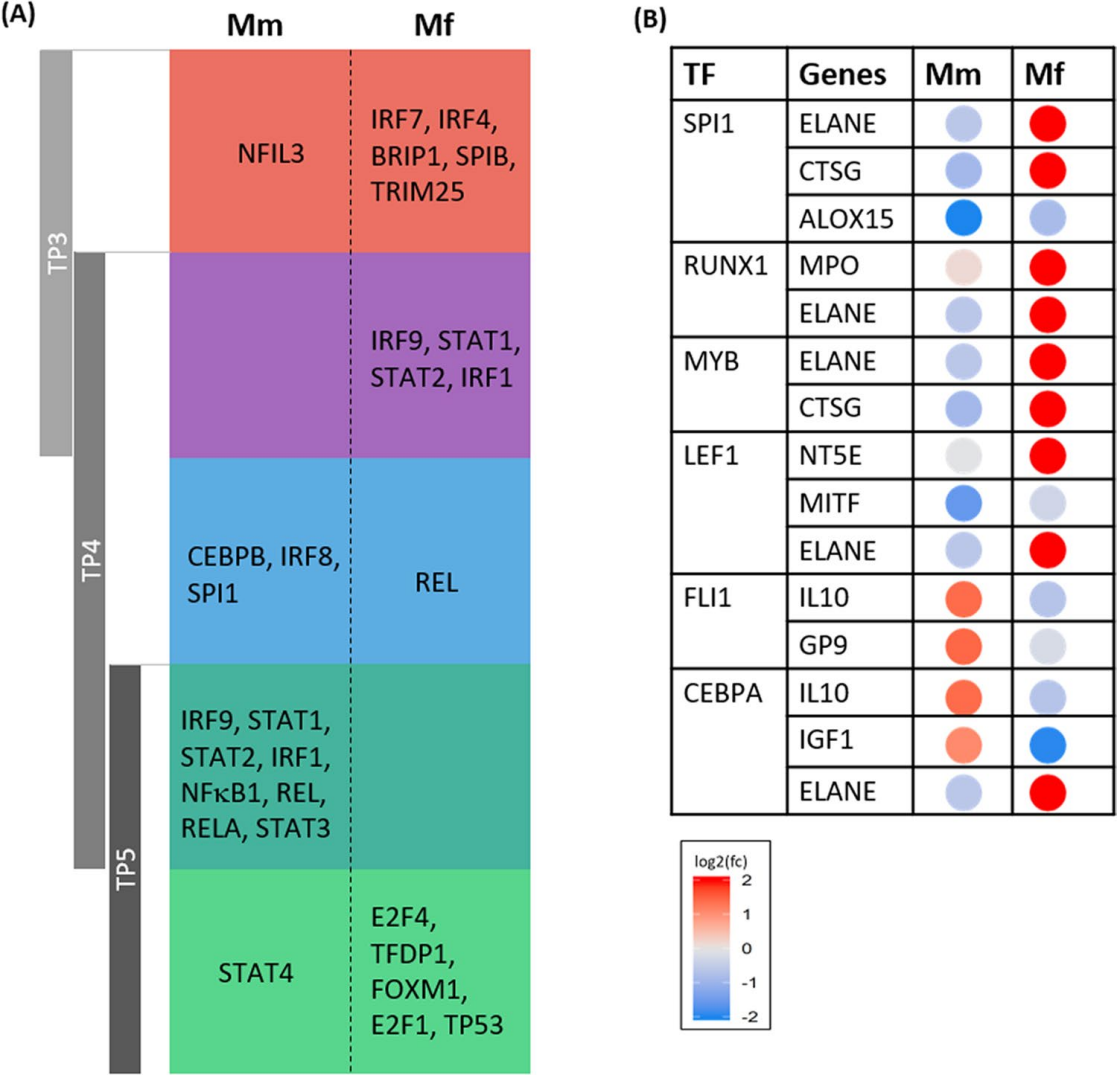
(**A**) Most significantly enriched TFs at each TP for the two host species. Mm has a disjointed set of TFs between TP3 and TP4 along with substantial overlap between TP4 and TP5. Mf, on the other hand, has substantial overlap between TP3 and TP4, which is then disjointed with TP5. (**B**) TFs enriched by DRGs at TP4 along with the corresponding DRGs. The heatmap shows differential expression of these genes at TP4 with respect to baseline.

**Figure 6:**
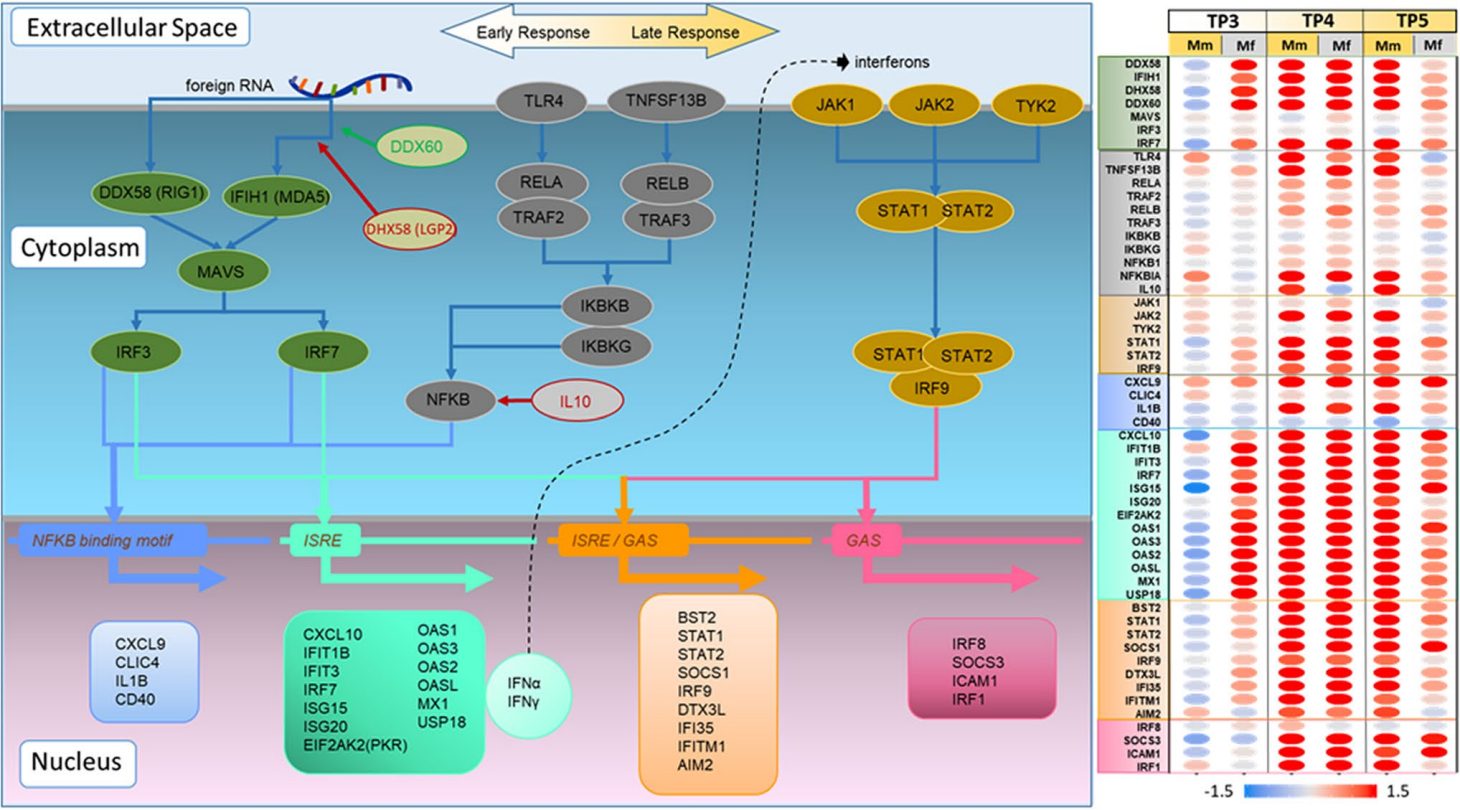
Transcription factors and associated genes of signaling pathways during the immune response. The TFs and genes shown are associated with differences in DEGs between early and late responses to infection. DEGs and their magnitudes are shown on the right for TP3, TP4, and TP5, with columns for each of Mm and Mf. Red dots signify up-regulation and blue dots down-regulation. In addition to DEGs at TP4 and TP5, the RIG1/MDA5-mediated PRR signaling pathway is included, as it is significantly different at TP3. IRF7-regulated genes expressed by ISRE also show significant changes. Finally, NFκB signaling, mediated by REL and RELA, plays a crucial role in controlling inflammation, and the ongoing strongly differential expression of the corresponding genes (ISRE- and GAS-regulated) at TP5 in Mm suggests a prolonged inflammatory response.

Consistent with the results presented above, the transcriptional data from Mf indicate significant enrichment for TF activity as early as TP3, and this response is absent in Mm (Figs. 5A,6). In particular, IRF7 and IRF4 are enriched in Mf at TP3 along with TRIM25, STAT2 and STAT1. In contrast to Mf, the corresponding Mm data only indicate slight enrichment of NFIL3 activity, which might suggest the emergence of precursors of common helper innate lymphoid cells; however, the signal is too weak to warrant a definitive claim.

Several other pertinent TFs are enriched at TP4, especially for Mm, where the parasitemia continues to rise unabated. In Mm, across TP4 and TP5, STAT3 is persistently activated, which is brought about by inflammatory cytokines. At least in humans, and presumably also in NHPs, enrichment of NFκB signaling related TFs—NFκB1, REL and RELA points toward the canonical NFκB signaling pathway^61^. The persistent activation of the anti-apoptotic pro-inflammatory NFκB pathway along with the opposing p53 pathway suggests that unduly extended inflammation might contribute to Mm’s severe, and indeed life-threatening systemic illness in response to the acute *P. knowlesi* blood-stage infection. Mm continues to show these immune responses at TP5 with additional activation of STAT4, a sign of IFN production by dendritic cells (DCs)^62^. In contrast, and paralleling the observations in gene sets, the immune response of Mf at TP5 is showing signs of recovery, with enrichment of E2F4, TFDP1, FOXM1, E2F1 and TP53, along with other TFs that are involved in balancing quiescence and cell cycle activation. The Mf furthermore enhances cell cycle related pathways by promoting both early stage (E2F1 and TFDP1) and late stage (FOXM1) cell cycle processes.

The intersection of TF sets at TP4 with those at TP3 and TP5 in both species (Table 1) distinguishes phases of infection in the two hosts. In Mf, TP4 shares many active TFs with TP3, but not TP5. This dramatic shift in TF profiles by TP5 in Mf could predictably allow the host to counteract the expansion phase of *P. knowlesi* by slowing down the inflammatory response and initiating recovery pathways. Mm, by contrast, essentially lacks an immune response at TP3. In Mm, TP3 also does not share any enriched TFs with TP4, whereas the sets of active TFs overlap substantially between TP4 and TP5, suggesting that the delayed TF program is sustained until TP5, resulting in continued inflammation and/or the lack of an appropriate immune response.

**Table 1:** Gene markers indicate enriched cell populations (and sub-populations) in the two species at different TPs during the infection.

	Mm	Mf
TP4	HSPC—Pre-B/NK	
	Monocyte—Non-classical	Monocyte—Non-classical
	B cell—Pro-B	
	NK cell—NKP	
TP5	Erythrocyte—ERY1, ERY/GRA2	Monocyte—Pre-Monocyte
	Nk cells—Cytokine NK	B cell—Cycling Pre-B
	Monocyte—Intermediate	NK cell—CLP
	Neutrophil—Meta-Myelocyte/Mature Neutrophil, Myelocyte	HSPC—G2M

HSPC: hematopoietic stem and progenitor cell; NK cells: natural killer cells; CLP: common lymphoid progenitor.

To highlight the main driving factors that differentiate the immune response in the two hosts at TP4, we identified the TFs enriched by the DRGs (Fig. 5B). These include TFs like RUNX1, SPI1, LEF1, FLI1 and CEBPA, which play important roles in hematopoiesis and lymphocyte differentiation. Interestingly, the affected genes suggest involvement of RUNX1, SPI1 and LEF1 in Mf while FLI1 and CEBBPA play a crucial role in Mm, including upregulation of IL10.

### Modular transcriptional repertoire analysis provides further insights into gene expression differences between the *Macaca mulatta* (Mm) and *Macaca fascicularis* (Mf) host species

**Figure 7:**
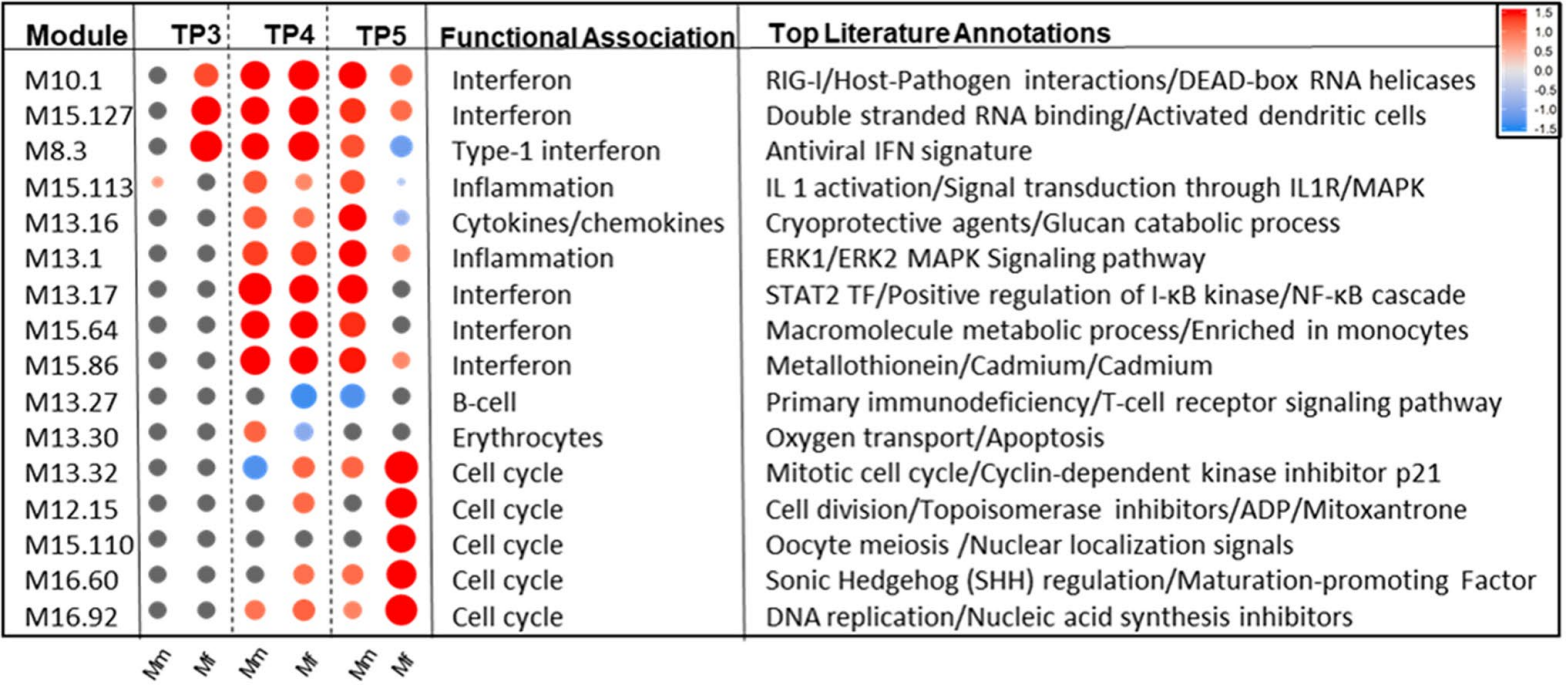
Heat map of most enriched transcription modules at each TP for both hosts. The heat map represents the enrichment score (ES) for each of the modules. Relevant information related to each module, for example pertaining to functional associations and literature annotations, is presented on the right; further details and the entire list of modules with enrichment is presented in the Supplements, including Table S6. The heatmap was created in the freely available programming language R.

The analysis of transcriptional modules yielded general agreement with our prior results, along with some surprising new insights (Figs. 7, S11–S12; Tables S4–S6). TP3 shows significant enrichment of IFN modules in Mf (M10.1, M15.127 and M8.3; Fig. 7). Type-1 IFN module M8.3 suggests induction of anti-pathogen effector genes like MX1 in addition to IFN modules M15.127 and M10.1, which may indicate immunopathology with host–pathogen interactions. M15.127 and M10.1 also point to a pathogen associated molecular pattern (PAMP), specifically, double-stranded RNA. Mm meanwhile shows a relatively modest enrichment of module M15.113, which is related to IL-1-mediated inflammation (Figure S11).

TP4 shows much similarity between the two hosts with respect to inflammation, IFN and cytokine-related modules (M13.16, M13.1, M10.1, M13.17, M15.127, M15.64, M15.86 and M8.3). However, some modules show distinguishing features. Down-regulation of the B-cell module M13.27 suggests possible inhibition of T cells with downregulation of CD96 and LY9. Among the erythrocyte modules, the difference in M13.30 suggests a relative difference in hematopoiesis by megakaryocyte erythroid progenitor markers like BLVRB, SLC25A39, HBM and HBQ1. The prostanoids module M8.2 suggests differences in platelet activation through enrichment of genes like PPBP, GP9, and others. Certain cell cycle related modules also suggest differentiating biological behaviors between the two hosts. Mm shows downregulation of a mitosis-related module (M13.32), while its upregulation in Mf suggests cell division.

Mf exhibits several enriched cell cycle-related modules that become even more significant at TP5 (M12.15, M13.32, M15.110, M16.60 and M16.92). At TP5, Mm still expresses enriched IFN modules that are now downregulated in Mf as compared to TP4. These enrichments align well with previous results showing that the inflammatory immune response in Mf subsides by TP5.

### Cell population markers

To complement these results, we analyzed single-cell reference gene markers to explore hematopoiesis and identify enriched cell types in blood samples (Table 1). These gene marker data indicate that non-classical monocytes are enriched in both hosts at TP4. Anti-inflammatory effects of non-classical monocytes include vascular homeostasis and are the first line of defense in terms of pathogen detection and clearance^63^. Mm shows enrichment of progenitor cells for both NK and B cells. By TP5, Mm exhibits enrichment of erythrocytes and neutrophils as well. The Mm may amplify cytokine signaling using NK cells. Activation of intermediate monocytes suggests their involvement in dealing with oxidative stress created by the infection. Interestingly, enrichment of hematopoietic stem and progenitor cells (HSPCs) in Mf is concentrated to the G2-M cell cycle phases suggesting proliferation of immature myeloid progenitors.

## Discussion

Our comparison between a disease-resilient host (Mf) and a highly vulnerable host (Mm), both infected with the same parasite species (*P. knowlesi*), was designed to yield insights into the host transcriptional programs associated with biological pathways that play significant roles in such infections. The identification and characterization of critical molecular and cellular differences between resilient and non-resilient hosts is an important step toward understanding the mechanisms of host-parasite responses.

The Mm and Mf species are separated evolutionarily by fewer than three million years and their geographical distribution areas overlap slightly (Figures S1, S2). We speculated that querying the evolutionary distance of homologous genes could potentially offer insights into the resilience of Mf. However, no correlation emerged in the two species between the sequence-level similarity of homologous genes and their differential response as parasitemia was rising. Interestingly, nevertheless, was the finding that the immune system-related DRGs are overrepresented in the outliers. While we performed a detailed analysis and interpretation of DRGs, a comprehensive evolutionary analysis might help identify key immune regulation checkpoints that should be queried in future work as they point to fundamental evolutionary differences in the immune responses of the two hosts.

Our analyses revealed that the immune system of Mf (but not Mm) senses the presence of foreign organisms as early as 3 dpi. Furthermore, prominent TFs generally associated with immune responses are solely activated in Mf by 3 dpi (Table 1). It is not surprising that Mf shares many common TFs with Mm at TP4, but it is interesting to note that prominent TFs generally associated with immune responses are solely activated in Mf as early as TP3 (Table 1). The first line of response in Mf is detection of infected hepatocytes via cytoplasmic PRR (cytokine) signaling via MDA5 and RIG1, which activates the TFs IRF3 and IRF7^64,65^. These signaling pathways activate the innate immune response, led by IFNα, which starts almost simultaneously with the pro-inflammatory response that is led by IFNγ^66,67^. In the context of responses to viruses, this type of detection is known to cause IFNα-mediated downregulation of viral genome replication^68^. Taken together, this early cytokine signaling along with IFN responses appears to be a crucial response signature in Mf that is missing or delayed in Mm, potentially rendering Mm more vulnerable to the infection (*cf.* Fig. 6).

Upregulation of cytoplasmic PRRs and the MDA5 signaling pathway in Mf at TP3 also marks the onset of a pro-inflammatory innate immune response led by IFNγ signaling. This response, along with regulation by certain transcription factors, including IRF7, STAT1, STAT2, and IRF4, leads to elevated cytokine production. IRF7 efficiently activates both IFN-α and IFN-β genes^69,70^ via the Interferon Sensitive Response Element (ISRE), which is clearly shown by corresponding gene expression that is activated in Mf at TP3 (Fig. 6). Interestingly, IRF7 has a short half-life (∼0.5–1 h)^71^ due to its susceptibility to ubiquitin-dependent degradation^72^. The labile nature of IRF7 may represent a mechanism critical to rendering the entire IFN gene-induction process transient, preventing overexpression of IFNs and harm to the host. Activation of the lymphoid-specific enhancer Spi-B transcription factor (SPIB) in the blood could either indicate the production of Type-1 IFN by plasmacytoid dendritic cells (pDCs) or IgM by mature B cells^73^, both of which have significant roles in the immune response.

The *P. knowlesi* infection transitions from hepatocytes to RBCs after about 5 days of parasite multiplication in the liver^32,33^. The following period of parasite multiplication in RBCs and the rising parasitemia are characterized by the most significant responses in terms of the number of DEGs in both species compared to baseline expression. All transcriptomic immune response signatures are consistent with the presence of pathogens in the blood, and judging by the small number of DRGs at TP4, both species launch a similar response. Against this background of similarity in transcriptomic responses, subtle differences are apparently very important.

In both species the rise in parasitemia at TP4 is marked by an elevated immune response via IFNα and IFNγ. The IFNγ-inducible genes are carefully orchestrated by the transcription factors STAT1, STAT2, and IRF9 to respond appropriately to the specific needs of the cell. This immune response is complemented by the IL-6 regulated JAK-STAT3 signaling pathway, which controls cytokines like erythropoietin, thrombopoietin and G-CSF and thereby may be involved in dealing with pathological conditions like anemia, thrombocytopenia, and neutropenia or, alternatively, the generation of antibodies through B-cell and plasma cell differentiation.

The innate immune system in the host spearheads the immune response not only by developing protective immunity but also by aiding the host in dealing with pathogenesis^74^, and the initial pro-inflammatory response attempts to clear the infection. However, when elevated and prolonged, inflammation leads to physiological deterioration and increasing severity of various pathological conditions. The fact that pro-inflammatory genes of the IFNγ pathway are more strongly elevated at TP4 in Mm than Mf suggests prolonged inflammation only in Mm, despite similar levels of parasitemia in both species at that time point. IL-10 is a chief anti-inflammatory receptor and affects various pathways to inhibit inflammatory cytokines like IL-6, IL-1 and TNFα. Substantial upregulation of IL-10 combined with enriched B-cell subpopulations (Pro B and Pre B) at this phase of infection is again suggestive of Mm’s attempt to stem the inflammation and fight the infection. In contrast, the presence of significantly enriched non-classical monocytes along with differentially expressed TNFα, IL1β and CCL3L3 is indicative of a direct response to the pathogen via the MyD88-MEK pathway^75^. Non-classical monocytes are known to produce more TNFα and less IL10, and timing of their enrichment might be crucial for the outcome.

Time point 5, representing the final days of high parasitemia, reveals the most dramatic differences between the species. Mm continues with its prolonged immune response with proinflammatory signatures, while Mf initiates a program of cell proliferation with the transcription of multiple genes involved in DNA replication and repair, mitosis, and cell cycle progression; all these suggest the onset of a recovery phase in the Mf. Enrichment of HSPCs in their G2M phase could support replenishment of lost cells and reinforcement of the immune response. Notably in Mf, the p53 pathway is significantly downregulated at TP5 in comparison to TP4, consistent with the goal of preventing p53 targets from hindering these cell proliferation pathways^76^. The gene TP53 is known to be activated in response to DNA damage and oxidative stress. It assists in apoptosis of damaged RBCs and maintains adult stem cell niches. In patients with malaria, TP53 has been shown to modulate inflammatory responses to infection^77^. Hypoxia activates this p53 signaling, and, indeed, hypoxia levels were higher in Mm than Mf, suggesting that higher hypoxia levels, sustained for a prolonged time, may contribute to the extended stress response of the p53 pathway. If so, the p53 pathway might be an important yardstick for inflammation. Sustained inflammation and delay in upregulation of cell development pathways in Mm is in fact revealing with regards to the mechanistic explanation for Mm’s deterioration, compared to Mf’s resilience and recovery, with the added major concern that infected Mm are rapidly running out of healthy, uninfected RBCs.

Unlike GSEA, which uses curated gene sets to define a function or process, the ‘modular transcriptional repertoire’ is derived from multiple (large-scale data) samples that display perturbed responses caused by various diseases or pathogens and pertain to a particular tissue (in our case white blood cells (WBCs))^78^. Analysis of whole modules rather than individual genes can increase our ability to detect interesting changes by decreasing the impact of multiple hypothesis testing and considering the inter-dependence of different transcript profiles. Interestingly, pre-patent phase modules in Mf are known to be associated with the detection of viruses (Table S6), especially influenza, which has frequently been confused with malarial infection due to early infection symptoms^79^. In line with duplicated efforts of IL10 and IL6 with respect to JAK-STAT signaling pathway, differential response of ERK1/2 MAPK signaling pathway related inflammatory modules suggests possible role of SOCS3 or STAT3 in extension of inflammation^80,81^.

The transcription differences between two closely related species, infected with the same parasite, offer hints to why one species faces severe life-threatening disease and requires aggressive treatment when infected with *P. knowlesi*, whereas the other becomes sick but rebounds without the provision of antimalarial drugs, as further detailed in Peterson et al*.*^51^. A noteworthy component of the difference appears to be the delayed detection of the parasites in Mm and the consequently delayed initial immune response in Mm, which comes too late for this species to recover.

Our analysis has identified interesting changes in molecular profiles between Mm and Mf. Not surprisingly, it also has shortcomings, which are by and large due to the infrequency of sampling in our longitudinal study, which in turn was dictated by regulatory blood draw limitations. Since most of the differentiating factors between the two macaque species point to the critical timing of pathogen detection and the timely switch of Mf’s transcriptomics program toward recovery, an iterative longitudinal study with denser sampling around these times would most likely allow more refined and definitive claims. Such experiments are warranted, particularly since our study results highlight promising prospects that one might use for the development of future anti-malarial treatments and vaccines. Many potential adjuvants for anti-malarial vaccines are under investigation, which mostly seek to target PRR signaling via TLR agonists^82^. Our analysis provides further mechanistic support for the application of such vaccines. During the later phases of the infection, IL10- and p53-related pathways could provide interesting drug targets. While it might be challenging to control IL10 due to its numerous roles, targeting p53 has already been demonstrated to attenuate malarial inflammation and protect from fever^77^.

Other comparative studies investigating *P. coatneyi* infections in the two macaque species, Mm and Mf, have shed light on differences in the pathology caused by this parasite species^83,84^. Unlike the current sporozoite-initiated infection study, however, these studies were based on the inoculation of blood-stage parasites. Raja et al. specifically indicate that cryopreserved *P. coatneyi* infected RBCs were obtained from the Centers for Disease Control and Prevention (CDC) and passaged once in intact (non-splenectomized) animals^83^. The *P. coatneyi* isolate (Hackeri strain) used in our published reports was confirmed by the CDC to have been passaged only in intact animals, where it caused disease including severe infections^40,85^, as documented previously^32,33,86^. The difference is important, because passage of *P. coatneyi* in splenectomized animals results in the circulation of schizonts^26^ that, according to electron microscopy, lack knob protrusions (unpublished data), suggesting the possibility of reduced *SICAvar* gene expression^87^, cytoadhesion, tissue sequestration, and virulence. Future comparative studies of *P. coatneyi*—an excellent model for *P. falciparum*—or *P. cynomolgi*—a model for *P. vivax*—that begin with sporozoites in the two macaque species would be of high interest.

Considering how closely related the two macaque species in our analysis are, one might have envisioned some simple switch in the expression of a few genes to result in the two disparate fates following *P. knowlesi* infection. Instead, our comparative analysis demonstrates that the differences in responses are rather subtle but widely distributed within a macaque species. In fact, the main and dramatic host species-specific difference we detected lies in the timing of responses, with Mf moving faster to identify the infection and trigger immune responses that can suppress the infection and support their recovery. This insight, if independently validated, is important for the development of potential new interventions as it suggests the need to search for means permitting the host to have enough time early on to generate a strong and effective immune response.

## Materials and methods

### Experimental setup and data collection

For this analysis, four male Mm and seven male Mf were infected with sporozoites of the Malayan strain of *P. knowlesi*^50^. The animals were observed daily during baseline periods, when inoculated with sporozoites, and throughout the infection. Female monkeys were excluded to avoid confounding effects of menstruation. Blood samples were collected at pre-defined TPs between 1 p.m. and 3 p.m. for both hosts, when the *P. knowlesi* cycle presents predominantly—if not exclusively—ring forms in circulation (Fig. 1). The Mf experiment underwent an initial unsuccessful sporozoite inoculation, and a new pre-infection baseline (TP2B) was established. The sporozoite re-inoculation was conducted approximately 80 days after the failed inoculation, and as shown in Figure S13, the failed inoculation did not have any apparent effect on the subjects and the observed transcriptomes. All pre-infection samples were used to establish baseline expression. The present study describes a secondary data analysis, while experimental details are described in Peterson et al*.*^51^. Nonetheless, all experiments involving NHPs were performed at the Yerkes National Primate Research Center (YNPRC), an AAALAC International-accredited facility. All methods were carried out in accordance with relevant guidelines and regulations. Specifically, all procedures followed ARRIVE guidelines and were approved by Emory’s IACUC and the Animal Care and Use Review Office (ACURO) of the US Department of Defense and followed accordingly. The Emory’s IACUC approval number was PROTO201700484—YER-2003344-ENTRPR-A.

After infection with sporozoites at Day 0, parasitemia in both species became patent 6 days post inoculation (dpi) (Fig. 1). Mf self-controlled the infection, while parasites in Mm kept rising as expected, with no evidence of the animals controlling the infection^51^. In the Mm species, untreated parasitemia could escalate rapidly such that the majority of all RBCs become infected, which would result in certain death of the animal. In our study, the monkeys were monitored carefully with blood smear readings taken twice daily during the acute stage of the infection and finally at 10 or 11 dpi when the animals had approximately 1% parasitemia and were euthanized for pathological measurements^51^. Red blood cell numbers and parasitemia levels are presented in Fig. 1 with details in Peterson et al*.*^51^. Other details of the experiments have been reported in publicly available databases: Mm (referenced as Experiment 06) at https://plasmodb.org/plasmo/app/static-content/PlasmoDB/mahpic.html and https://www.ncbi.nlm.nih.gov/bioproject/524357, and Mf (referenced as Experiment 07) at https://plasmodb.org/plasmo/app/static-content/PlasmoDB/mahpic.html and https://www.ncbi.nlm.nih.gov/bioproject/526495. Additionally, a clinical and histopathological analysis of these cohorts can be found elsewhere^51^. The transcriptomics data for both hosts can be found in the Gene Expression Omnibus (GEO accession numbers: GSE127079, GSE128115). PlasmoDB and NCBI-Bioproject are public databases that are freely accessible to anyone without specific permission.

### Orthology analysis and gene similarity scores

The reference Mm genome, corresponding transcripts and annotations were obtained from Zimin et al.^88^. The data can be downloaded from the reference site^89^. The corresponding reference genome files for Mf were obtained from NCBI annotation release 101^90^ and can be downloaded from the NCBI ftp server^91^.

Nucleotide sequences from the corresponding transcripts (fasta) file were used to detect reciprocal best hits. This correspondence was achieved with a reciprocal BLAST protocol described in reference^92^ and used to identify orthologous transcripts. Unfortunately, the reciprocal-best-hits method does not guarantee orthology and is prone to shortcomings like its handling of gene duplications. Thus, to estimate the similarity between two orthologous sequences, transcripts differing in length by more than 50 bp were removed to avoid manual curation. Additionally, transcripts with less than 85 percent identity were removed. Finally, to calculate the evolutionary distance between homologous genes, a robust and widely accepted metric of sequence similarity was used^93^. The similarity score for the transcripts was calculated using BLAST alignment scores. These scores are calculated by assigning a value to each pair of nucleotides and then summing these values^94^. These scores were then normalized by the lengths of the transcripts to obtain the similarity scores for each pair of homologous genes.

### Read mapping and gene expression analysis

Samples were sequenced using Illumina Hi-seq 3000. For each host, the reads were mapped using STAR (version 2.5.2b)^95^ against corresponding references (*cf.* sources in Orthology analysis above). For each species, a composite reference genome was assembled using STAR index, and further raw RNA-seq reads were mapped to the combined reference using STAR.

Raw reads were normalized for library size, sequencing depth and composition using the DESeq2^96^ standard library size normalization method (estimateSizeFactors function). Custom R scripts were used to implement the DESeq2 normalizations and create PCA plots using variance stabilized transformation (vst function) of the normalized data. The major genes contributing to each PC were extracted using the largest absolute values of components for the eigenvectors for each PC. The enrichment *p*-value for each gene set was calculated using the top 200 genes in a hypergeometric test.

### Differential expression and differential response

Differential expression of genes was calculated using DESeq2^96^. First, we filtered low-abundance genes by removing genes which had low read counts in more than 20% of the samples, as is standard procedure. For differential expression analysis, the samples were modeled using species as the major factor and infection-TPs as a secondary factor for a subset of samples for each host. For differential responses, a species:infection-TPs interaction term was added to all the samples.

Since DESeq2 models the expression data as a negative binomial distribution, dispersion was estimated using the ‘estimateDispersions’ function, and differential expression was calculated using Wald’s test (nbinomWaldTest). DESeq2 functions adjust the *p*-value using the Benjamini–Hochberg method. For differential expression analysis, data were contrasted on the TP infection state with respect to baseline. For differential responses, the samples were contrasted on the interaction term. Representative examples of differentially expressed and differentially responding genes are highlighted in Figure S5. The DESeq2 package in R was used^97^.

### Gene set enrichment analysis

Differentially expressed genes were analyzed for the enrichment of gene sets using the GSEA toolkit (version 3.0)^98^ by the Broad Institute. The gene sets used for the analysis were Hallmark^55^ and Gene Ontology (GO)^99,100^. The pre-ranked GSEA module of the toolkit was used for the analyses, where genes were ranked by an adjusted *p*-value and the sign of the fold-change.

We performed the ranked analysis in two ways. First, to retain only robust signals, we selected genes that were significant (adjusted *p* < 0.01 and log2(fold-change) > 1) and used their ranked list. Second, for comparisons between infection TPs between species, we selected all genes and used their ranked list. This step reduced biases in enrichment of gene sets as the same genes would be present in each set and the enrichment score (ES) would be calculated by their ranks. The toolkit calculates an ES for each of the gene sets that demonstrates overrepresentation of the gene set at the top/bottom of the ranked gene list. GSEA uses a weighted standard Kolmogorov–Smirnov statistic to calculate the ES. To account for different sizes of gene sets and correlations between gene sets and expression data, a normalized ES was calculated by considering 1000 permutations of ES, calculated by randomly assigning phenotypes to samples. Finally, false positives were restricted by applying a false discovery rate (FDR) correction^101^ and using the threshold FDR < 0.25.

To elucidate the transition between TP4 and TP5 further, ranked gene set analysis was performed (Fig. 4). All gene sets identified as Hallmark and GO Biological Processes were ranked based on their normalized enrichment scores (NES) for each species at both TP4 and TP5. For Hallmark sets (Figs. 4C,D), a rank flow plot was created to visualize changes in ranks between TP4 to TP5 for each species. For GO sets (Fig. 4E), we refined the approach to identify which among the important gene sets undergo a transition between TP4 and TP5 for Mf but remain essentially unchanged in Mm. We achieved this by first calculating the rank difference for each gene set from TP4 to TP5 as shown by a violin plot. Each data point in the distribution represents a GO gene set and the quantitative value (y-axis) is the rank change (Rank @TP4—Rank @ TP5). We narrowed our analysis to the most significant gene sets (rank < 200 for at least one species/TP). We then analyzed the gene sets which remain almost unchanged in Mm (absolute rank difference < 100) but are significantly changed in Mf (absolute rank difference > 1000). This filtering resulted in lists of gene sets that were then summarized by removing redundant GO terms using REVIGO^102^. The results are summarized in blue and red boxes in Fig. 4E.

GO-Net^103^ and REVIGO^102^ were also used in Figs. 4E, S6, S8 and S9 to summarize GO results and use their hierarchical structure for inference. Custom R scripts were created to plot heatmaps and bar plots for GSEA results; the scripts are available in the github package binf.gsea.visualizations at (https://github.com/LBSA-VoitLab/packages). To analyze the hierarchical structure of GO annotations, we used the Cytoscape (v 3.4) plugin Bingo (v 3.0.3)^104^ and the treemaps were formed using REVIGO^102^.

### Upstream targets and motifs

Transcription factors and upstream regulators were analyzed with iRegulon^59^ and TRRUST db^60^. While iRegulon predicts TF-targets using ChIP-seq data, the TRRUST database is built on highly curated TF-target associations acquired from the literature. Cumulative results of both applications were used. In some cases, the results were quite diverse, thereby leading to a larger list of TFs (e.g., for Mf at TP3).

The iRegulon (v1.3) plugin for Cytoscape (v3.4) was used to predict TFs and gene set motifs. iRegulon implements a genome-wide ranking and recovery approach to detect enriched TFs and motifs. It looks for cis-regulatory sequences among co-expressed genes. For this particular analysis, we used the 10 K PWMs (position weight matrices) database with NES > 3 and FDR < 0.001 on motif similarity.

TRRUST uses a sentence-based text mining approach and is very well curated. Customized R scripts and packages were created to analyze and merge the results and then create a regulatory network; the scripts are available in the binf.trrust github package at https://github.com/LBSA-VoitLab/packages.

### Modular transcriptome repertoire

The third generation of the modular transcriptional framework was used as described in Altman et al*.*^105^. Module-level details such as transcripts and annotations were obtained from the corresponding supplementary material of Altman et al*.*^105^. We identified 382 uniquely annotated modules, but in some cases more than one module had a similar functional annotation. A ranked list using differential expression for each host species followed by enrichment analysis of each of these modules was established using the method explained above for GSEA^101^. The modules that were considered for this analysis had *p* < 0.01 (adjusted) and FDR < 0.25. The scripts are available in the binf.modular github package at https://github.com/LBSA-VoitLab/packages.

### Cell population markers

To gauge the changes of various cell populations, we performed enrichment analysis of these populations and subpopulations. The Marker Gene database from the Atlas of Human Blood Cells^106^ was used to compile cell markers for various cell populations. This database consists of 43 transcriptional cell clusters that are profiled from single-cell deep sequencing. Ranked lists of DEGs were used for enrichment, which was calculated using the method explained above for GSEA. The database was downloaded from the source and custom scripts were created to analyze and obtain enrichment scores, as detailed in Subramanian et al.^98^. A cutoff adjusted *p*-value of 0.05 was chosen to select enriched cell populations.

## Supplementary Information

Supplementary Information.

## Data Availability

Details of the experiments have been reported in publicly available databases: Mm at https://plasmodb.org/plasmo/app/static-content/PlasmoDB/mahpic.html and https://www.ncbi.nlm.nih.gov/bioproject/524357; Mf at https://plasmodb.org/plasmo/app/static-content/PlasmoDB/mahpic.html and https://www.ncbi.nlm.nih.gov/bioproject/526495. The transcriptomics data for both hosts can be found in the Gene Expression Omnibus (GEO accession numbers: GSE127079, GSE128115). Supplementary tables and R packages for data processing and visualizations can be found at https://github.com/LBSA-VoitLab/Mm_Mf_analysis and https://github.com/LBSA-VoitLab/packages.
