# Supplementary material for: Dramatic transcriptomic differences in Macaca mulatta and Macaca fascicularis with Plasmodium knowlesi infections
Source: Sci Rep. 2021 Sep 30;11:19519. doi: 10.1038/s41598-021-98024-6 (PMC8484567; doi:10.1038/s41598-021-98024-6)
Supplement: Supplementary file 1 — Supplementary Information. [file 41598_2021_98024_MOESM1_ESM.docx]

**Supplements:**

**Dramatic Transcriptomic Differences in *Macaca mulatta* and *Macaca fascicularis* with *Plasmodium* *knowlesi* Infections**

**Anuj Gupta, Mark P. Styczynski, the MaHPIC Consortium, Mary R. Galinski, Eberhard O. Voit, Luis L. Fonseca**

These supplements contain additional figures and tables, as cited in the main text.


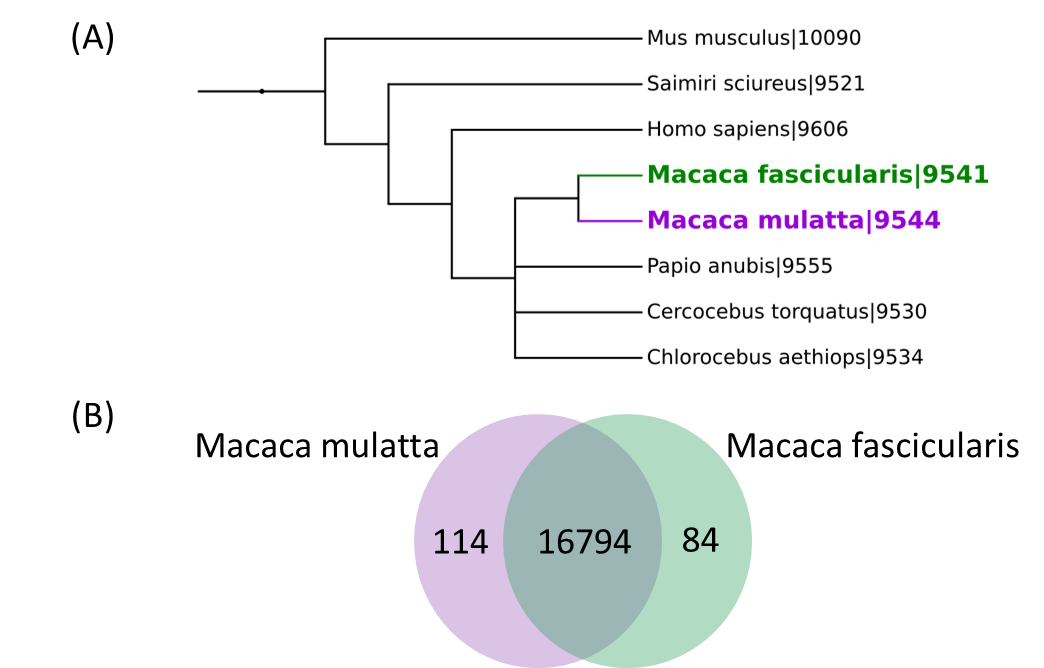


**Figure S1: Overall genomic similarity and evolutionary separation of *M. mulatta*, *M. fascicularis*, and related species.** A: The phylogenetic tree was assembled using the phylot web tool <https://phylot.biobyte.de/>. The sources for this information can be found on the following website: <https://ori.hhs.gov/education/products/ncstate/primate.htm>. B: Venn diagram of homologous genes between Mm and Mf. OrthoVenn (<http://aegilops.wheat.ucdavis.edu/OrthoVenn/>) was used to create this diagram.


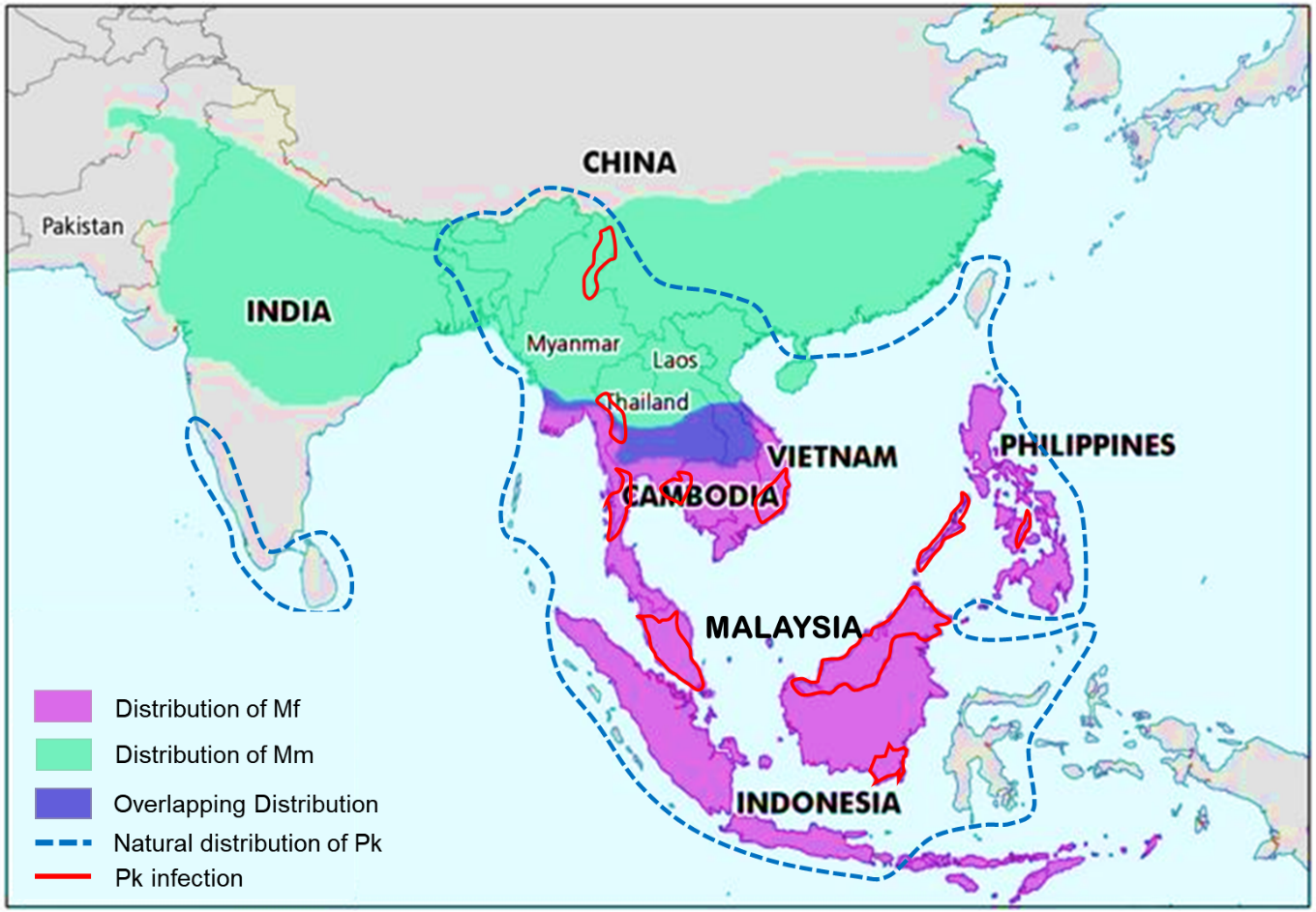


**Figure S2: Distribution of *M. mulatta* and *M. fascicularis* macaques and the *Plasmodium knowlesi* pathogen.** Regions of geographical distribution of Mm and Mf, overlaid with areas where *P. knowlesi* (Pk) is present and able to infect the macaques (dashed blue lines). Areas with observed *P. knowlesi* infections are marked with red lines. This image was adapted from (1), with additional information from (2) and (3) added, using the free programming language R. Article (1) is an Open Access article governed by the terms of the Creative Commons Attribution License (<https://creativecommons.org/licenses/by/2.0>), which permits unrestricted use, distribution, and reproduction in any medium, provided the original work is properly cited.

Street, S. L., Kyes, R. C., Grant, R. & Ferguson, B. Single nucleotide polymorphisms (SNPs) are highly conserved in rhesus (Macaca mulatta) and cynomolgus (Macaca fascicularis) macaques. *BMC Genomics* **8**, 480, doi:10.1186/1471-2164-8-480 (2007).

Moyes, C. L. *et al.* Defining the geographical range of the Plasmodium knowlesi reservoir. *PLoS Negl Trop Dis* **8**, e2780, doi:10.1371/journal.pntd.0002780 (2014).

Singh, B. & Daneshvar, C. Human infections and detection of *Plasmodium knowlesi*. *Clin Microbiol Rev* **26**, 165-184, doi:10.1128/CMR.00079-12 (2013).


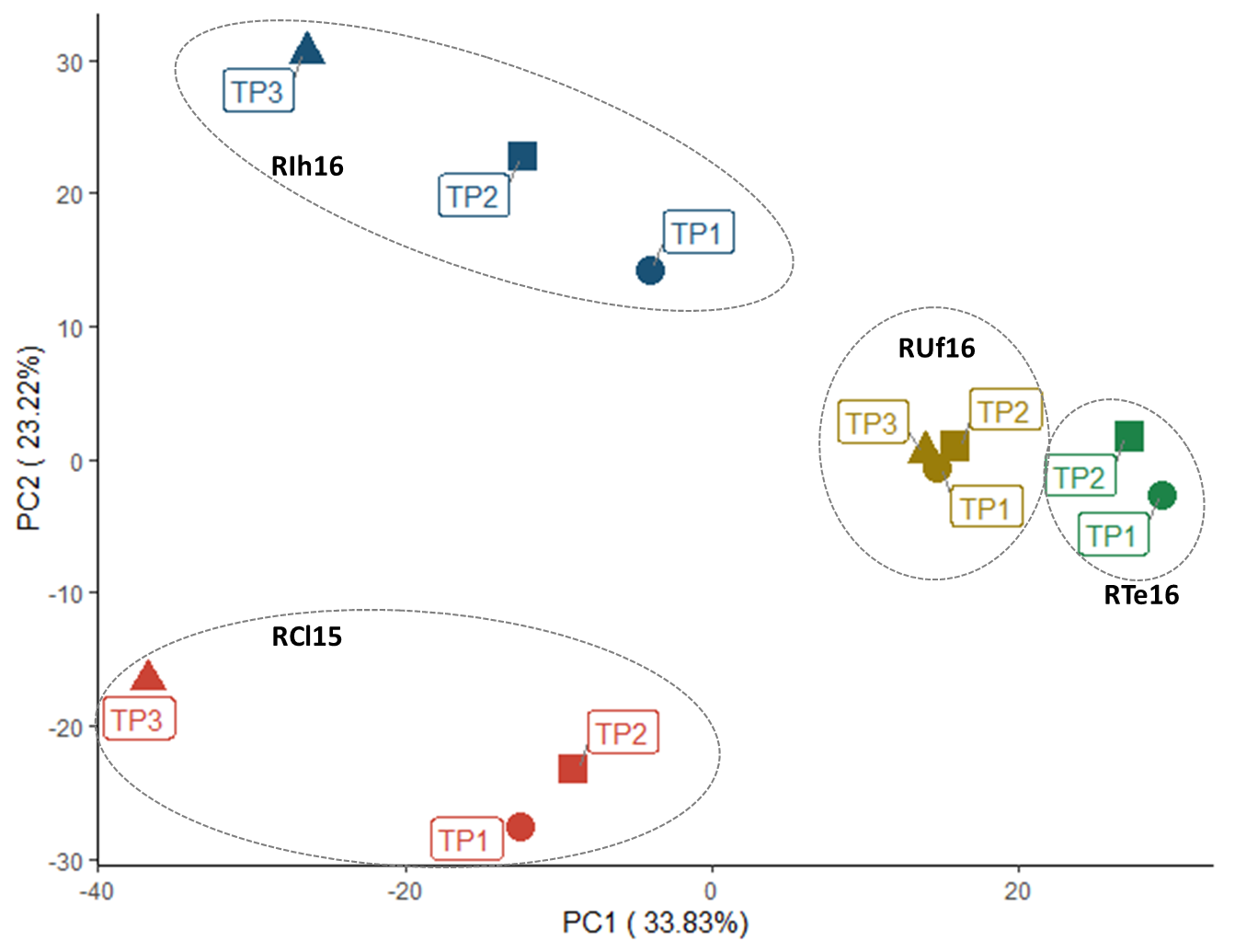


**Figure S3: Principal component analysis (PCA) of *M. mulatta* at three time points.** Whole blood samples were taken for transcriptomics analysis of Mm before (TP1 and TP2) and soon after infection (TP3). Any potential differences in TP3 transcriptomics with respect to baseline are dominated by subject-specific variance, which is evident from the clustering of individual subjects (ellipses). RCL15, RTe16, RUf16 and RIh16 represent codes of the macaques from which the blood samples came for this analysis. TP3 data are missing for RTe16. The figure was created with the software R.


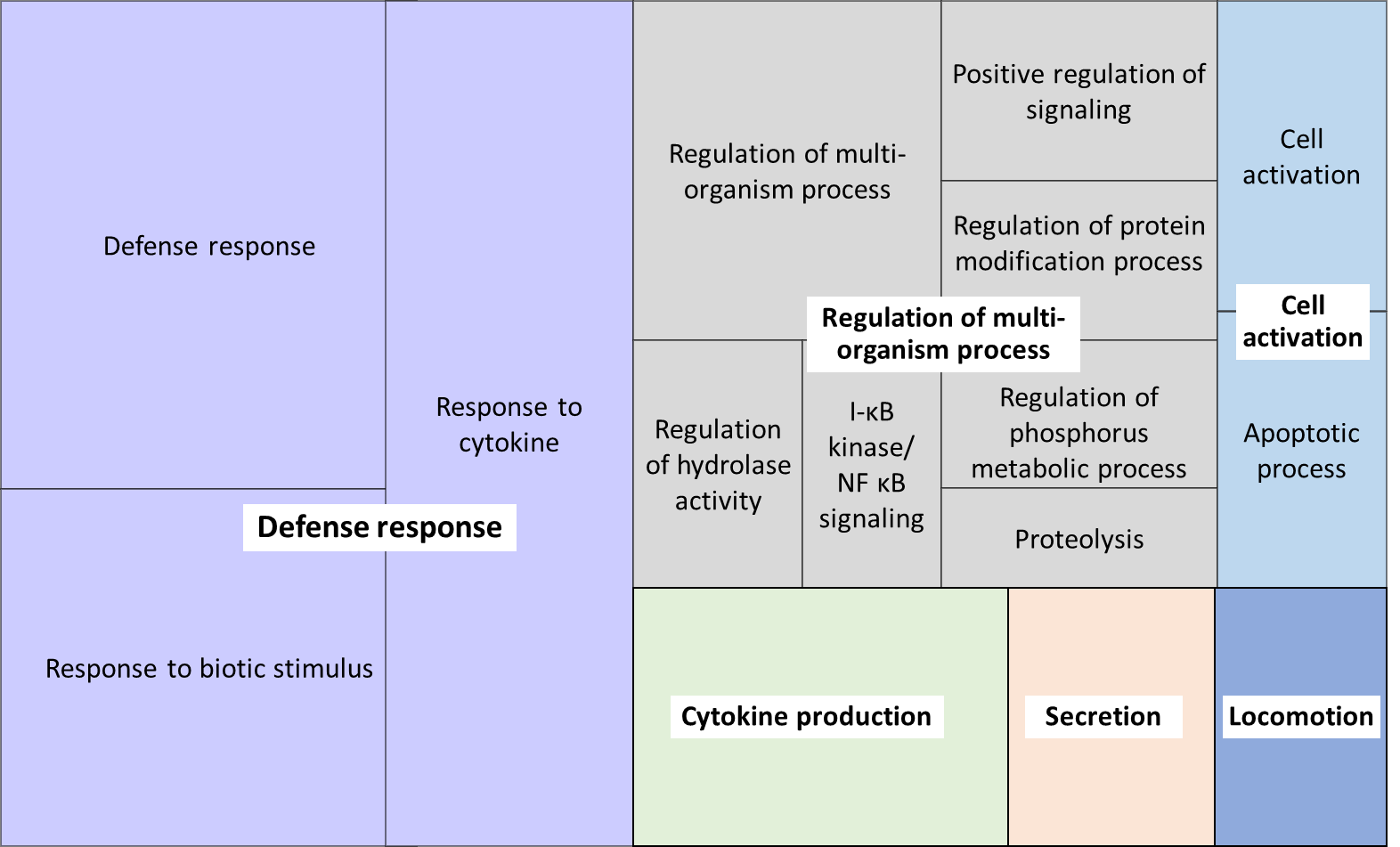


**Figure S4: REVIGO tree map showing top GO annotations from PC2 of the PCA in Figure 2.** The size of each rectangle is proportional to the –log10(*p*-value) with bigger rectangles representing higher significance. A generic defense response, response to biotic stimuli and response to cytokines are the most significant GO annotations. This visualization summarizes the large set of enriched GO annotations and removes redundant GO annotations, grouping them based on the GO term hierarchy. The figure was created with the software R.


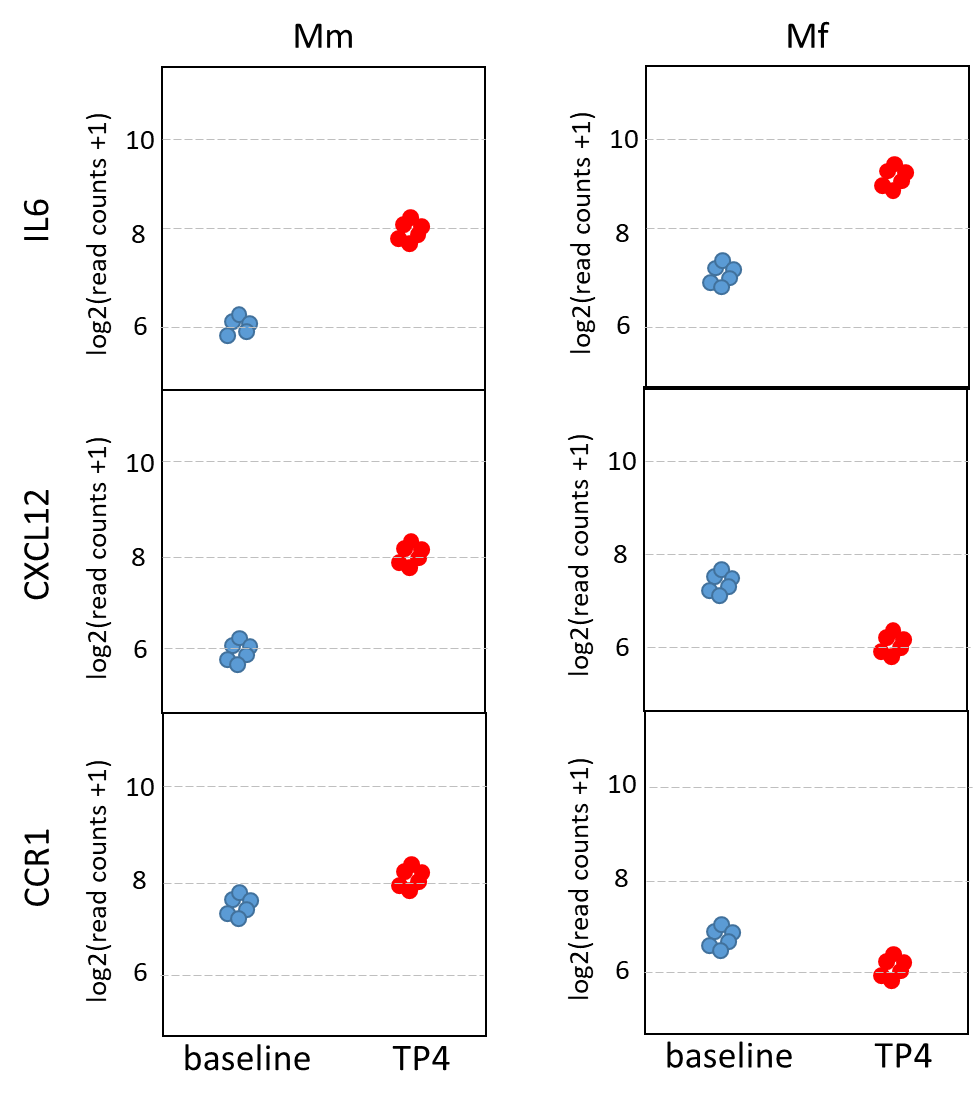


**Figure S5: Example plot explaining DEG *vs*. DRG (Note: These are not actual values; they are presented to highlight the importance of DRGs).** IL6 is differentially expressed in Mm and Mf but is not differentially responding (values at TP4 higher than baseline in both species). CXCL12 is differentially expressed in Mm and Mf, and it is also differentially responding. Expression of CCR1 is not significantly different in Mm and Mf but the species are differentially responding, as the (insignificant) changes in each species are in opposite directions. The figure was created with the software R.


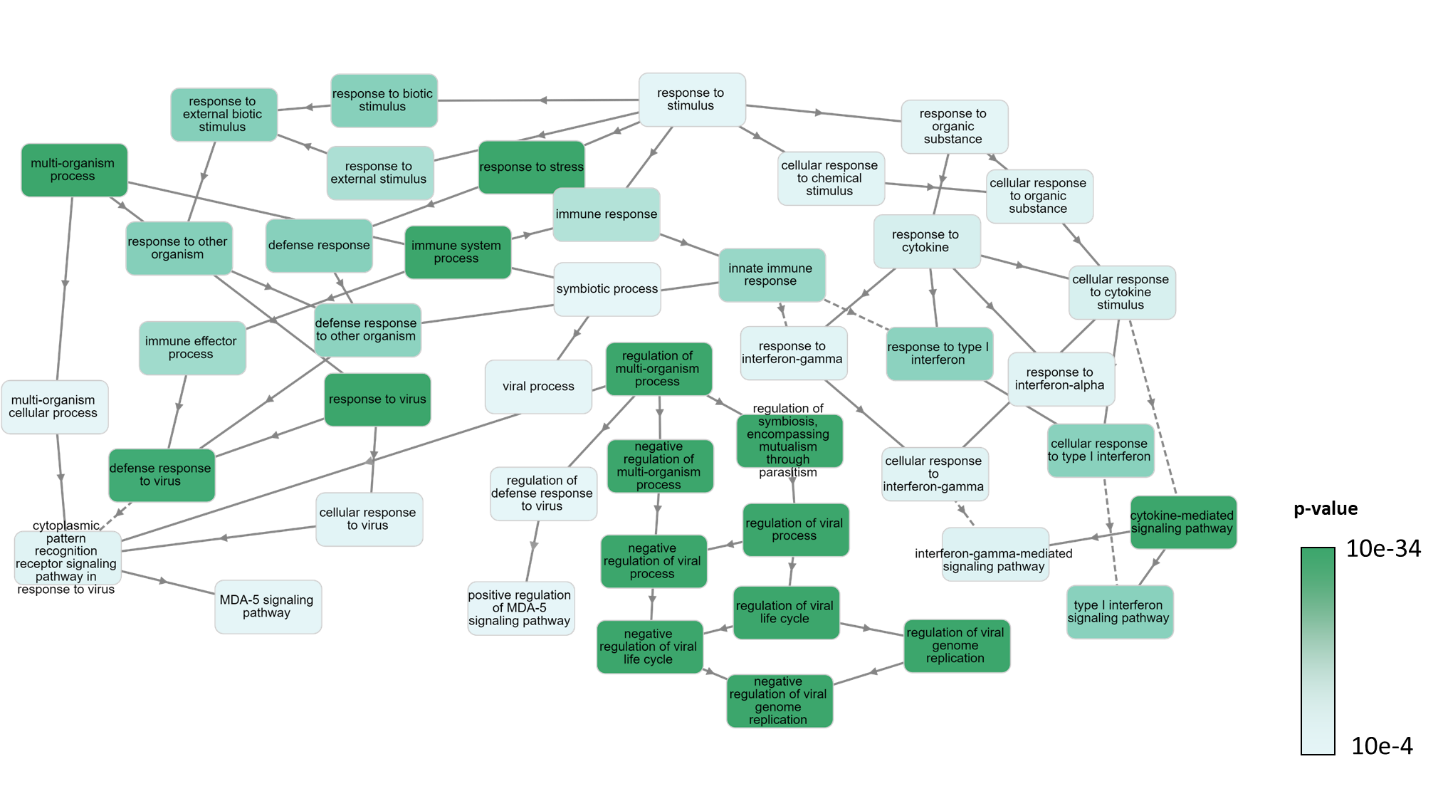


**Figure S6: Hierarchical graph for GO annotations significantly enriched in Mf at TP3 (*p* <0.001).** GO gene-sets offer the advantage of inherent hierarchy, which adds structure and groupings to enriched pathways. The highly enriched gene-sets (*p* < 0.0001) are colored, with darker colors reflecting higher enrichment. The connecting directional arrows show the hierarchy. At TP3, Mf shows a strong defense response directed against viruses or other invading organisms. The graph was created with the GO-Net web application ([https://tools.dice-database.org/GOnet/](https://tools.dice-database.org/GOnet/jobdceebb10-0c4b-45cf-b686-086331cbf2d2/result)).


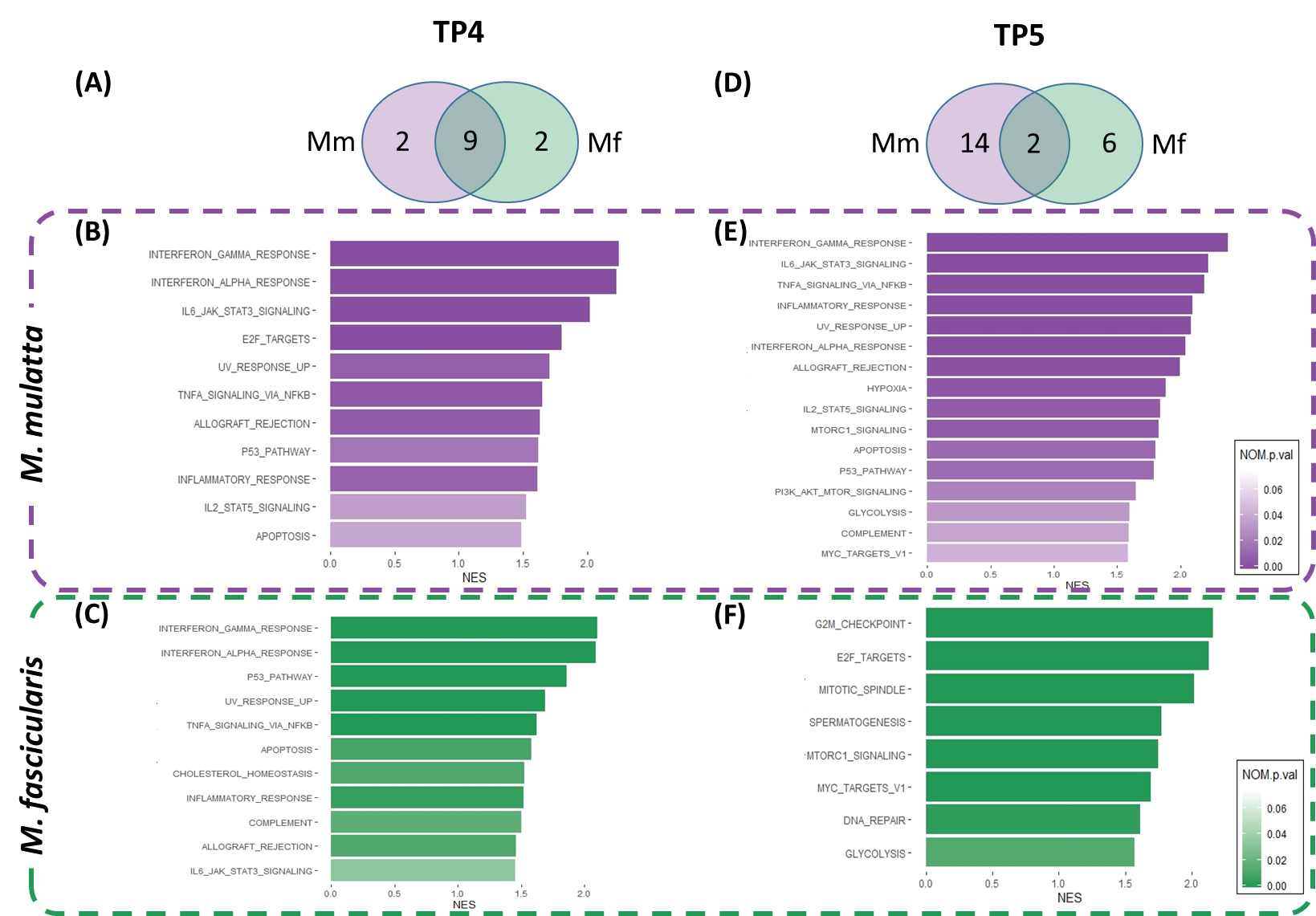


**Figure S7: Results of GSEA for TP4.** **(A):** Venn Diagram showing shared Hallmark pathways between Mm and Mf at TP4 (FDR< 0.25, table S2). **(B, C):** Bar plots showing significantly enriched GO annotations from GSEA of Mm(B) and Mf(C) at TP4. **(D):** Venn Diagram showing shared Hallmark pathways between Mm and Mf at TP5 (FDR< 0.25, Table S3). **(E, F):** Bar plots showing the significantly enriched GO annotations from GSEA of Mm(E) and Mf(F) at TP5.

X axis: Normalized Enrichment Scores (NES) colored, colored by significance. The figure was created with the software R.


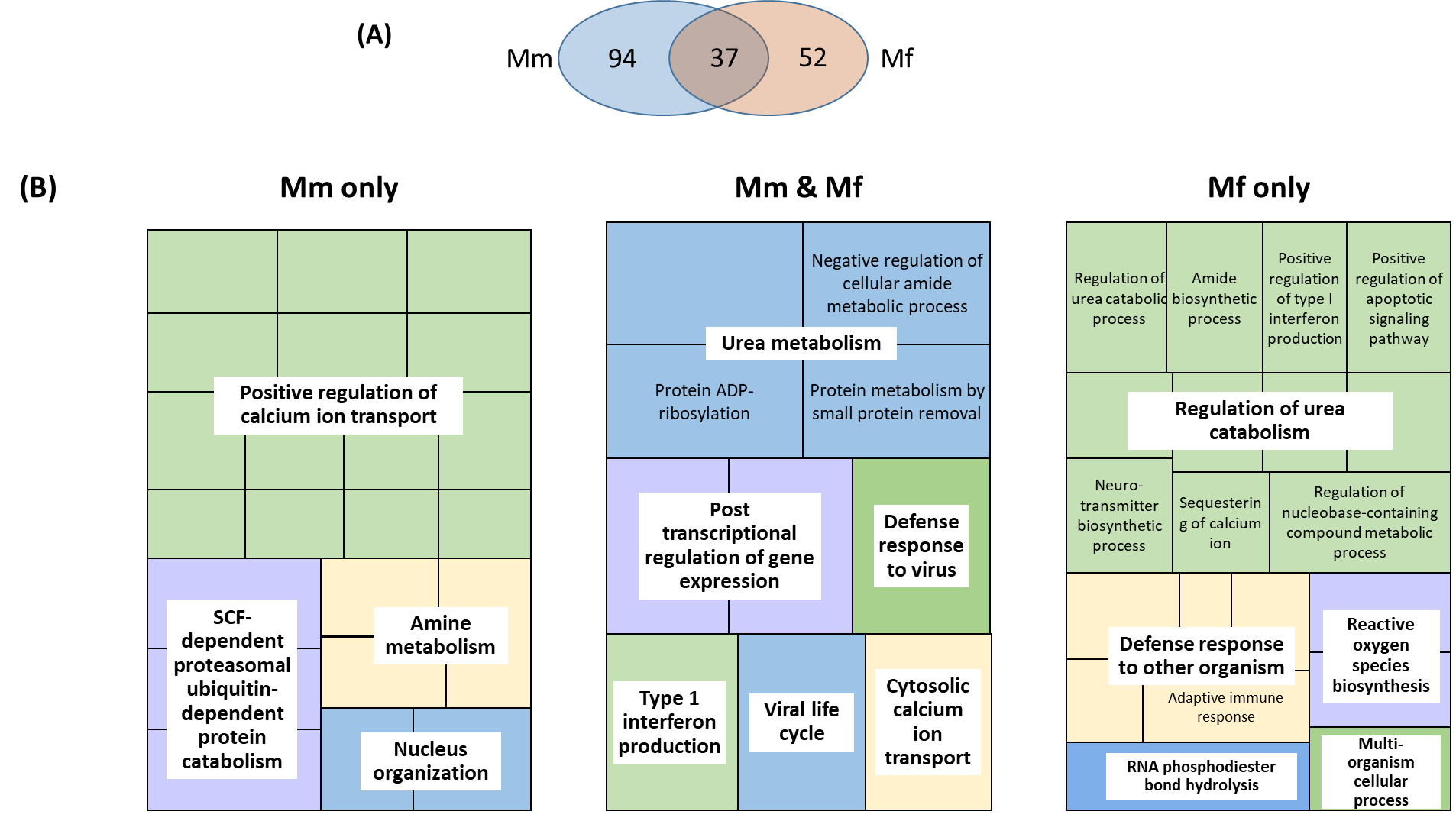


**Figure S8: Most highly enriched pathways at TP4. (A)**: The Venn diagram shows numbers of common and different most highly enriched GO Biological Processes in Mm and Mf at TP4. **(B)**: Reduced tree maps of enriched GO processes for each group (Mm only, Mm and Mf, Mf only). The GO annotations were summarized using REVIGO to remove redundant annotations for each set. The figure was created with the software R.


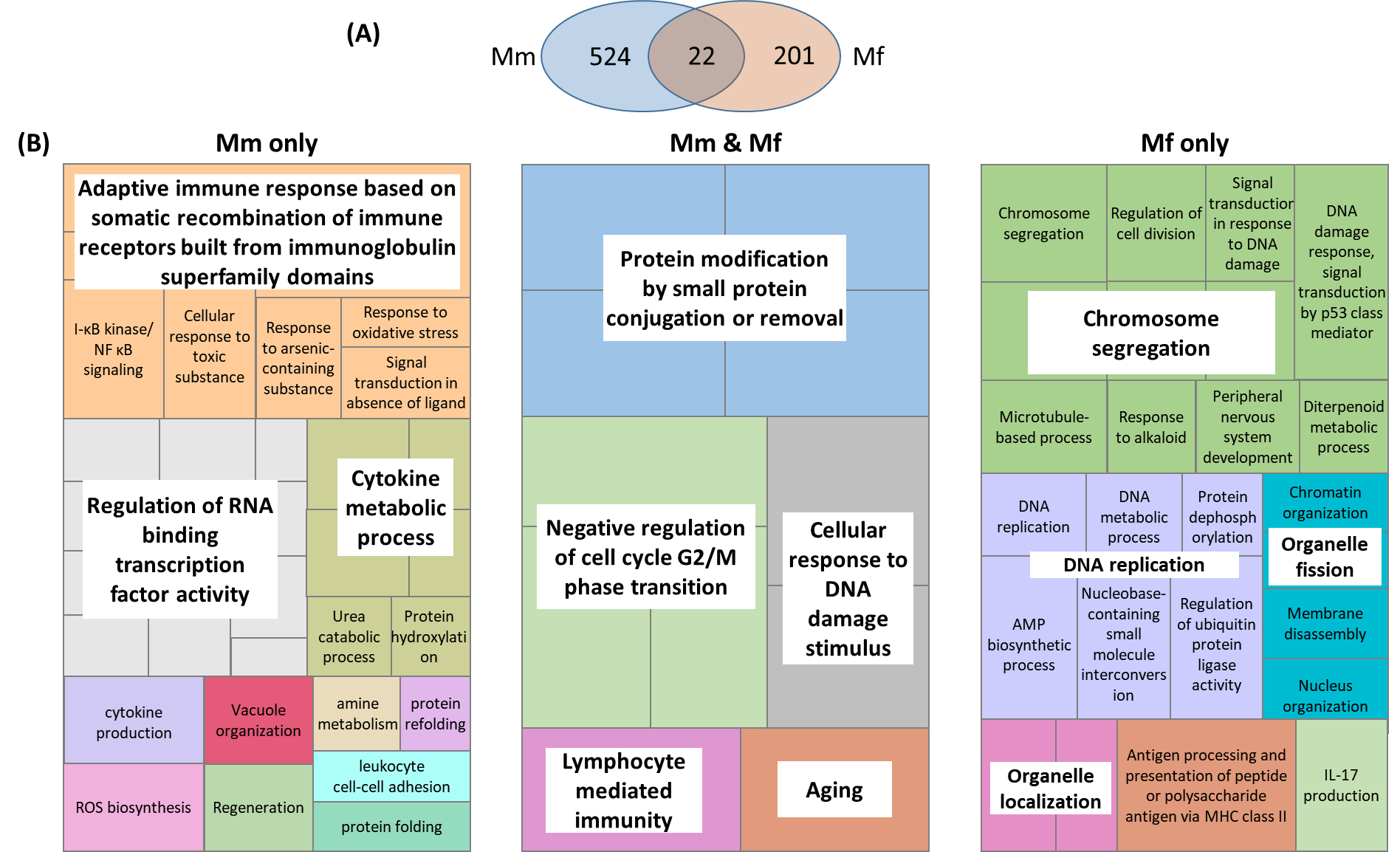


**Figure S9: Most highly enriched pathways at TP5. (A):** The Venn diagram shows the numbers of common and different most highly enriched GO Biological Processes of Mm and Mf at TP5. **(B):** Reduced tree maps of enriched GO processes for each group (Mm only, Mm and Mf, Mf only). The GO annotations were simplified using REVIGO to remove redundant annotations. The figure was created with the software R.


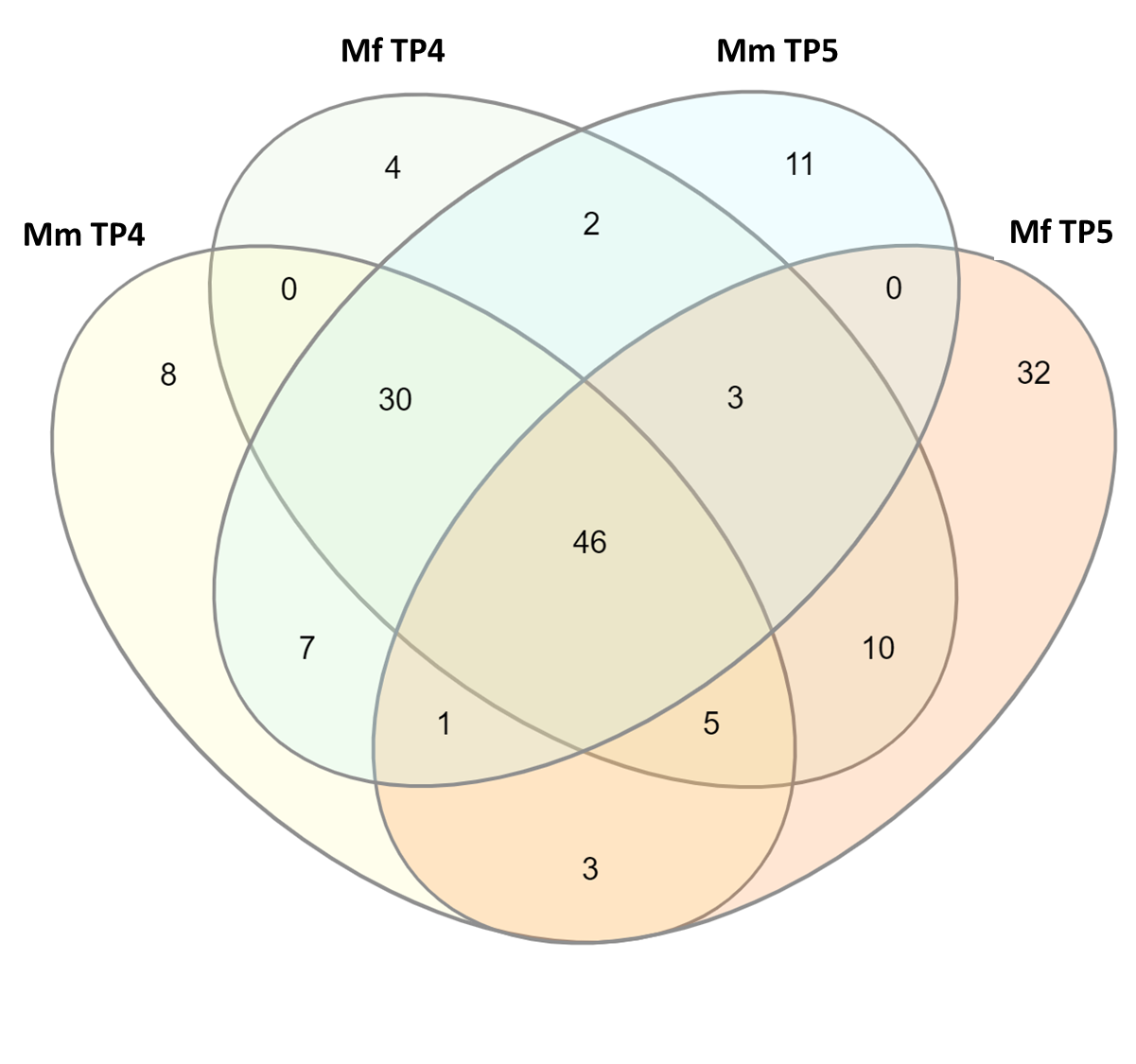


**Figure S10: Venn diagram top 100 GO annotations at TP4 and TP5 for Mm and Mf.** The intersections of these GO-annotated gene-sets visualize the similarity among the different groups. The majority (76) of pathways are common between Mm at TP4, Mm at TP5 and Mf at TP4. By contrast, Mf at TP5 shows the highest mutually exclusive gene-sets (32), indicating a stark deviation from TP4. The figure was created with the software R.


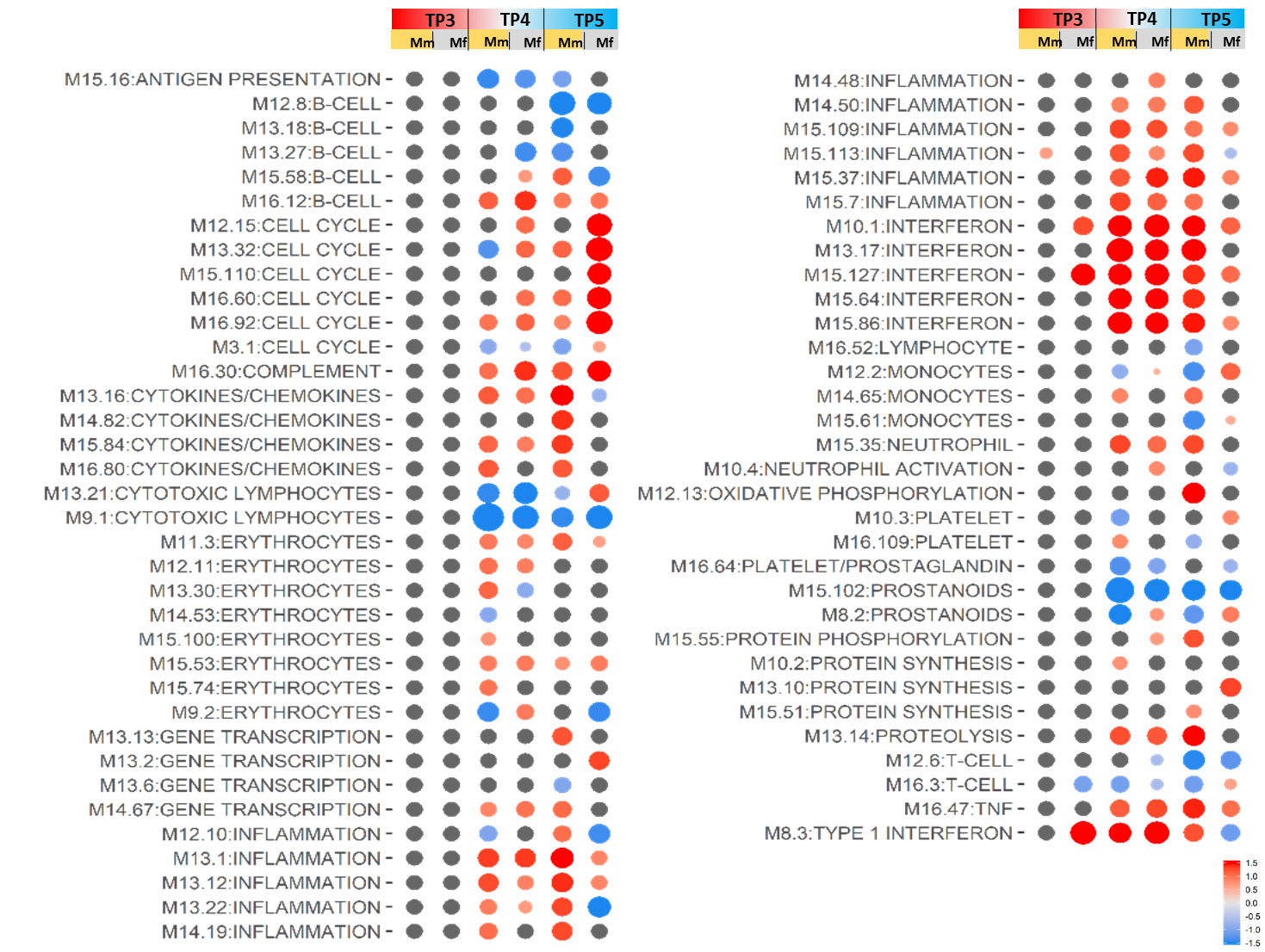


**Figure S11: Comparison of changes in main transcriptome modules of Mm and Mf at TP3, TP4 and TP5.** The modular transcriptome analysis presented in the form of a heat map. The modules can be associated with a functional annotation, with details presented in Table S7. The image consists of all modules that are not as strongly enriched as those in Figure 7. The figure was created with the software R.


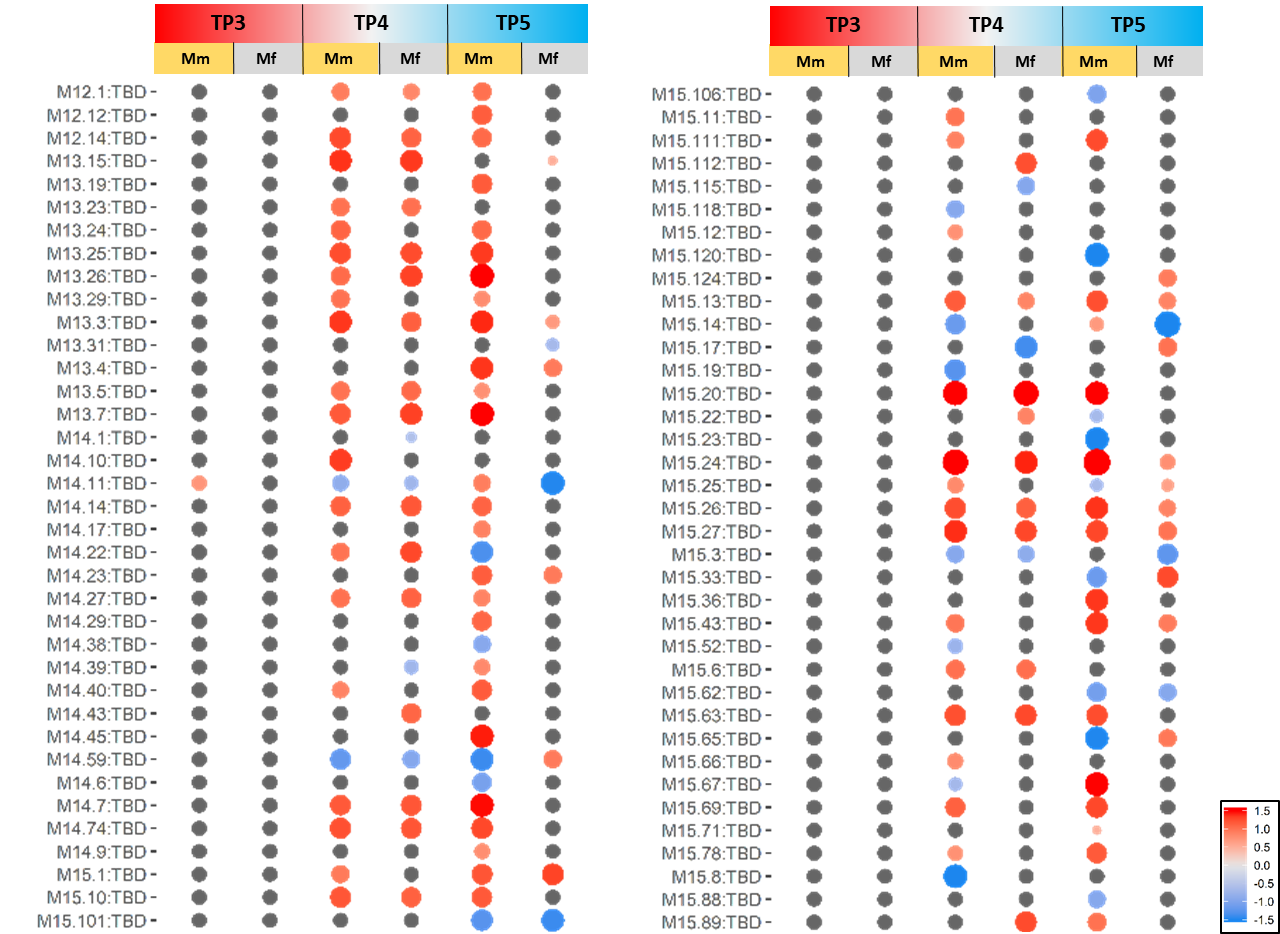


**Figure S12: Heat map of modular transcriptome analysis comparing Mm and Mf at TP3, TP4 and TP5**. A functional annotation could not be associated with these modules but additional information regarding association with diseases, as well as enrichment in KEGG and GO can be found in Table S8. The figure was created with the software R.

**Figure S12** (continued)


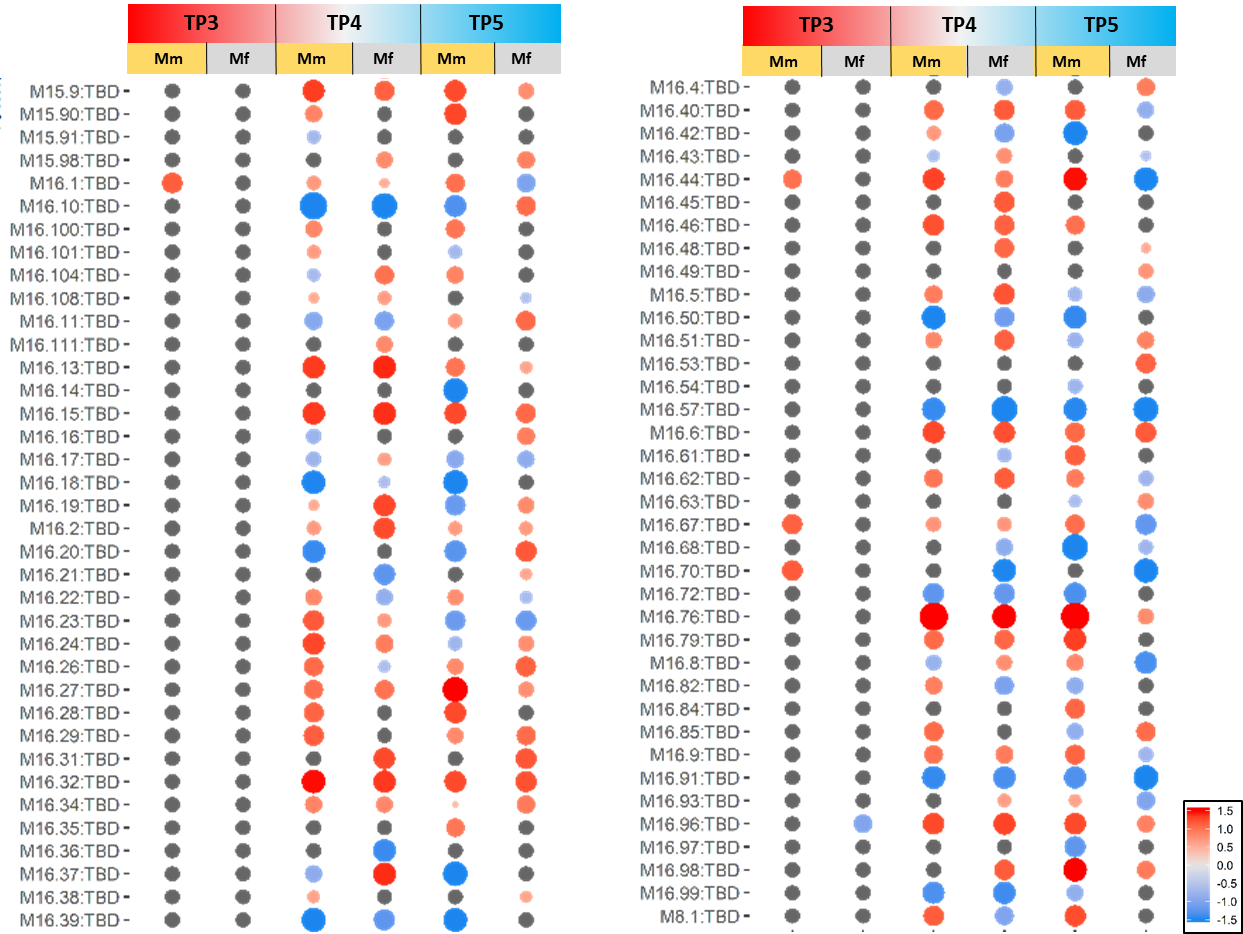


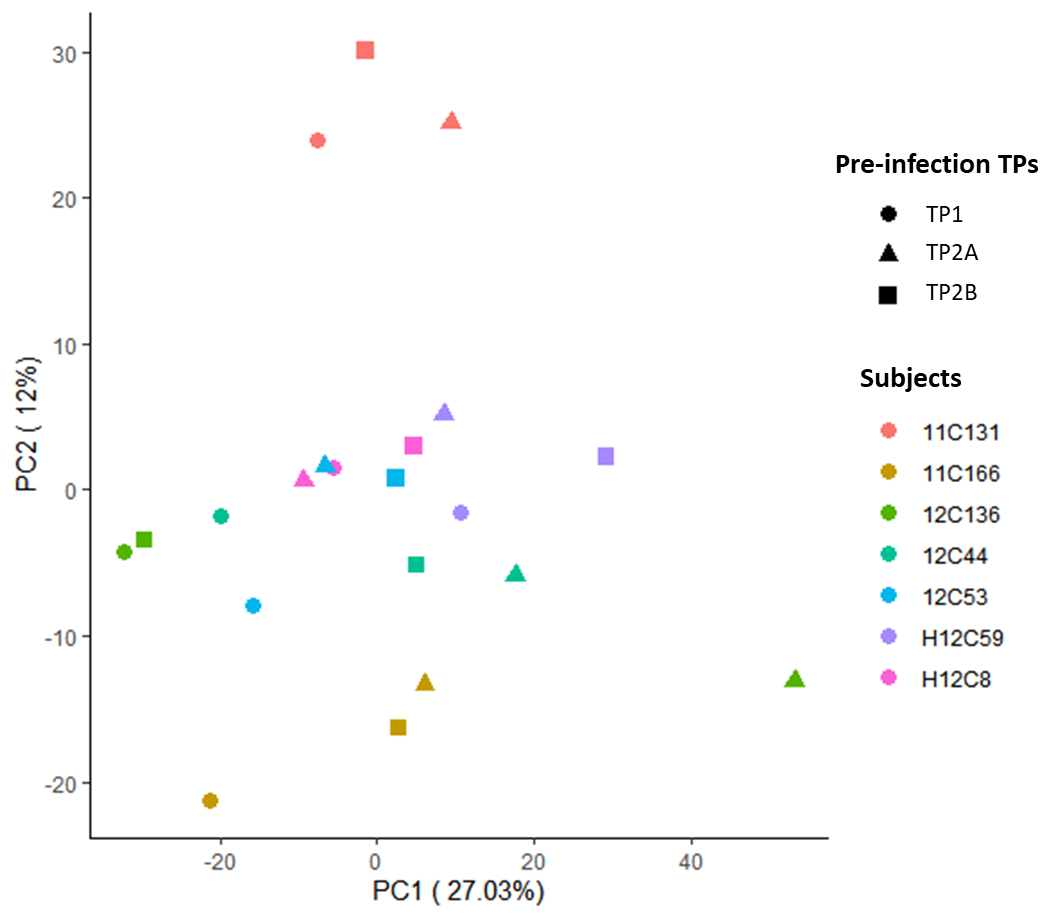


**Figure S13: PCA of pre-infection samples for Mf**. Samples from individual subjects cluster together. TP2A and TP2B do not show any noticeable difference due to failed inoculation. The figure was created with the software R.

**Table S1**: GSEA using Hallmark gene sets of Mm and Mf at TP3, TP4 and TP5. Gene sets in orange-shaded boxes have positive enrichment scores; *i.e.*, they were upregulated, while teal-shaded have negative enrichment scores.

**Table S2:** GSEA using hallmark gene sets for Mf *vs*. Mm at TP3, TP4, and TP5

**Table S3:** Transcription factors for differentially expressed genes, according to iRegulon, with normalized enrichment scores (NES), for the two macaque species at different time points.

**Table S4**: Modular transcriptome. Highly enriched and functionally annotated modules, complementary to Figure 7

| **Module ID** | **Module functional association title** | **Disease activity up** | **Disease activity down** | **Top literature lab annotations** | **Top matched pathway in KEGG** | **Top GO term Biological Processes** |
| --- | --- | --- | --- | --- | --- | --- |
| M10.1 | Interferon | HIV, *Burkholderia*, SoJIA, RSV, Influenza, SLE, TB, Transplant |  | RIG-I/Host-Pathogen interactions/DEAD-box RNA Helicases | RIG-I-like receptor signaling pathway | Response to virus |
| M15.127 | Interferon | HIV, SOJIA, RSV, Influenza, SLE, TB, Transplant |  | 2',5'-oligoadenylate/ Orthomyxoviridae infections/ Double stranded RNA |  | Immune response |
| M8.3 | Type-1 interferon | HIV, SoJIA, RSV, Influenza, SLE, TB, Transplant |  | Transcriptome/Transcriptome/Orthomyxoviridae Infections |  | Immune response |
| M15.113 | Inflammation | Staph, *Burkholderia*, SoJIA, TB |  | Antirheumatic agents/ IL-1/ IL -1α | MAPK signaling pathway | Intracellular signaling cascade |
| M13.16 | Cytokines/chemokines | Staph, *Burkholderia*, SoJIA, Influenza, TB |  | Cryoprotective agents/Nicotinic acids/Glycerol | Starch and sucrose metabolism | Glucan catabolic process |
| M13.1 | Inflammation | Staph, *Burkholderia*, SoJIA |  | Mitogen-activated protein Kinase 1/Mitogen-activated protein kinase 3/ERK1 | Acute myeloid leukemia | Regulation of cell proliferation |
| M13.17 | Interferon | HIV, Burkholderia, SOJIA, RSV, Influenza, SLE, TB |  | STAT2 Transcription Factor/ NLR proteins/ Inflammatory bowel diseases |  | Positive regulation of I-kB kinase/NF-kB cascade |
| M15.64 | Interferon | HIV, *Burkholderia*, SoJIA, Influenza, SLE, TB |  | Tripartite motif proteins/ Systemic lupus erythematosus/Thymine |  | Immune response |
| M15.86 | Interferon | HIV, *Burkholderia*, SoJIA, RSV, Influenza, SLE, TB |  | Metallothionein/Cadmium/Cadmium |  |  |
| M13.27 | B-cell | MS | Kawasaki, TB, SoJIA, Pregnancy, Staph, *Burkholderia* | Lymphocyte specific protein tyrosine kinase p56(lck)/ZAP-70 protein-tyrosine kinase/LCK | Primary immunodeficiency | Positive regulation of immune system process |
| M13.30 | Erythrocytes | RSV, Transplant, Melanoma | HIV | Biotransformation/Biotransformation/Gases |  | Oxygen transport |
| M13.32 | Cell cycle | HIV, RSV, SLE, Kawasaki |  | Aurora kinase/Cyclin A2/Cyclin-dependent kinase inhibitor p21 | Cell cycle | Mitotic cell cycle |
| M12.15 | Cell cycle | HIV, SLE, Kawasaki |  | Topoisomerase inhibitors/ADP/Mitoxantrone |  | Mitosis |
| M15.110 | Cell cycle | MS, HIV, RSV, SLE |  | Polo-like kinase/Protein sorting signals/Nuclear localization signals | Oocyte meiosis | Cell cycle |
| M16.60 | Cell cycle | HIV, RSV |  | Cyclin B1/Cyclin B/Maturation-promoting factor | Oocyte meiosis | Mitosis |
| M16.92 | Cell cycle | HIV, RSV, SLE |  | Aurora kinases/ Nucleic acid synthesis inhibitors | DNA replication | DNA metabolic process |

**Table S5**: Modular transcriptome. All functionally annotated modules that are complementary to those in Figure S11.

The complete table can be found at: <https://github.com/LBSA-VoitLab/Mm_Mf_analysis/blob/master/Tables/table_S5.txt>

A representative sample of the table is as follows:

| **Module ID** | **Module functional association title** | **Disease Activity Up** | **Disease Activity Down** | **Top Literature Lab Annotations** | **Top matched pathway in KEGG** | **Top GOTERM Biological Processes** |
| --- | --- | --- | --- | --- | --- | --- |
| M15.16 | Antigen presentation |  | B-Cell deficiency, SoJIA, Staph, *Burkholderia* | Dendritic Cells/Antigen-presenting cells |  | positive regulation of peptidase activity |
| M12.8 | B-cell |  | Influenza, HIV, B-Cell Deficiency, TB, SoJIA, Pregnancy, Staph, *Burkholderia* | Antibodies, Monoclonal, Murine-Derived/Antigens, CD20/Antibodies | B cell receptor signaling pathway | B cell activation |
| M13.18 | B-cell | MS | Pregnancy, Staph, *Burkholderia* | Environmental Pollutants/Toxic Actions/Lung Neoplasms | Spliceosome | RNA processing |
| M13.27 | B-cell | MS | Kawasaki, TB, SoJIA, Pregnancy, Staph, *Burkholderia* | Lymphocyte Specific Protein Tyrosine Kinase p56(lck)/ZAP-70 Protein-Tyrosine Kinase/LCK | Primary immunodeficiency | positive regulation of immune system process |
| M15.58 | B-cell |  |  | Sulfur compounds/Gene expression regulation, Enzymologic/Biomarkers, Tumor |  | molting cycle process |
| M16.12 | B-cell |  |  | Genome/Mutation/Genome Components |  | protein localization |
| M12.15 | Cell cycle | HIV, SLE, Kawasaki |  | Topoisomerase Inhibitors/ADP/Mitoxantrone |  | mitosis |

**Table S6**: Modular transcriptome. All modules that do not have a functional association in Figure S12.

The complete table can be found at: <https://github.com/LBSA-VoitLab/Mm_Mf_analysis/blob/master/Tables/table_S6.txt>

A representative sample of the table is as follows:

| **Module ID** | **Module functional association title** | **Disease Activity Up** | **Disease Activity Down** | **Top Literature Lab Annotations** | **Top matched pathway in KEGG** | **Top GOTERM Biological Processes** |
| --- | --- | --- | --- | --- | --- | --- |
| M12.1 | TBD | MS | Kawasaki, B-Cell Deficiency, Pregnancy, Staph, *Burkholderia* | Mitochondria Apoptotic/Mitochondria/Cell Respiration |  | translation |
| M12.12 | TBD | MS, SoJIA, JDM |  | Peroxides/Oxidative Stress/Superoxide Dismutase | Proteasome | negative regulation of macromolecule metabolic process |
| M12.14 | TBD | SLE | RSV | Intestinal Neoplasms/Colonic Diseases/Colorectal Neoplasms |  |  |
| M13.15 | TBD | Staph |  | Protein Domains/Protein Structural Elements/Protein Interaction Domains and Motifs | Endocytosis | intracellular signaling cascade |
| M13.19 | TBD | SOJIA | RSV, Transplant | Protein Domains/Protein Structural Elements/Phosphoinositide | Prostate cancer | regulation of transcription |

**Table S7:** Evolutionary similarity score calculated for homologous genes between Mm and Mf.

The complete table can be found at: https://github.com/LBSA-VoitLab/Mm_Mf_analysis/blob/master/Tables/table_S7.txt

A representative sample of gene similarity score for some genes:

| **Genes** | **Similarity (max 1)** |
| --- | --- |
| A1CF | 0.998385361 |
| A2M | 0.990088106 |
| A2ML1 | 0.994613313 |
| A4GALT | 0.989350913 |
| A4GNT | 0.990607389 |
| AAAS | 1 |
| AACS | 0.968049155 |
| AADAC | 0.988264361 |
| AADACL2 | 0.99402787 |
| AADACL3 | 0.979161436 |
| AADACL4 | 0.986658196 |
| AADAT | 1 |
| AAED1 | 0.99751861 |
| AAGAB | 0.982069481 |
| AAK1 | 0.999382462 |
| AAMDC | 0.994035785 |
